# Minimally Invasive Surgical Techniques for Renal Cell Carcinoma with Intravenous Tumor Thrombus: A Systematic Review of Laparoscopic and Robotic-Assisted Approaches

**DOI:** 10.3390/curroncol32050256

**Published:** 2025-04-28

**Authors:** Yiting Wu, Shuyang Feng, Ping Fu

**Affiliations:** 1 Department of Nephrology, Kidney Research Institute, West China Hospital, Sichuan University, Chengdu 610041, China; wuyiting_97@stu.scu.edu.cn; 2 Department of Urology, West China Hospital, Sichuan University, Chengdu 610041, China; fengshuyang@stu.scu.edu.cn

**Keywords:** renal cell carcinoma (RCC), intravenous tumor thrombus (IVTT), minimally invasive surgery, laparoscopic surgery, robotic-assisted surgery, oncological safety, systematic review

## Abstract

Introduction: Locally advanced renal cell carcinoma (RCC) with intravenous tumor thrombus (IVTT) represents 4–10% of renal tumors. This review assesses the safety and outcomes of minimally invasive techniques, specifically laparoscopic (LAP) and robotic-assisted (RA) methods, for treating RCC with IVTT. Methods: A literature search across several databases identified 54 studies (42 case series, 12 cohort studies) for analysis. Perioperative outcomes, including operative time, blood loss, transfusion rates, length of stay, and complications, were compared based on IVTT levels. Results: LAP and RA techniques were feasible for low-level IVTT, showing similar perioperative results. RA outperformed LAP in high-level IVTT with shorter operative times and lower blood loss and transfusion rates, despite managing more complex cases. RA maintained stable cancer-specific mortality (CSM) and metastasis rates, whereas LAP exhibited higher rates in high-level cases. Both techniques had low local recurrence rates. Conclusion: RA may be a superior option for RCC with IVTT, especially in high-level cases, but the data come mainly from specialized centers, signaling a need for multicenter validation and standardized criteria. Long-term outcomes require further study to assess RA’s non-inferiority to LAP.

## 1. Introduction

Locally advanced renal cell carcinoma (RCC) with intravenous tumor thrombus (IVTT) accounts for 4–10% of renal tumor patients [1]. A recent study reported that the improved treatment of advanced renal cell carcinoma appears to be responsible for the survival rise, with a rate of stage III RCC of 8.3% during 2004–2015 reported by the National Cancer Data Center [2].

Aggressive surgical management, including radical nephrectomy (RN) and intravenous tumor thrombectomy (IVTTx), is recommended for well-selected patients with locally advanced RCC and IVTT by the latest guidelines from the European Association of Urology (EAU). Although with different details, the current National Comprehensive Cancer Network (NCCN) clinical practice guidelines also prefers this surgical principle [3]. It is widely accepted that the extent of IVTT basically determines the choice of surgical approach and technique [3,4].

Surgical management for RCC patients with tumor thrombus has always been one of the challenges in the field of urology. Once, open surgery was the only default approach for RCC with VTT [5]. Nevertheless, along with the considerable advances in laparoscopic and robotic techniques, minimally invasive surgery has emerged as an acceptable option for complicated RCC patients due to its minimally invasive feature. Meanwhile, due to its specific ethics and paucity of control, almost all evidence on minimally invasive techniques for these patients is rather heterogeneous and of a low level, which makes the optimal patient selection criteria for minimally invasive surgery still to be elucidated [6].

Diverse minimally invasive techniques applied to RCC with IVTT have emerged dramatically, and are boldly combined with technologies from other departments, including perioperative renal artery embolization, IVC filters, and even cardiopulmonary bypass [7]. In addition, thanks to researchers’ pioneering techniques for laparoscopic and robotic management of renal tumor with high-level tumor thrombi, the indications of minimally invasive surgery for these patients are expected to be further expanded.

While laparoscopic and open surgical approaches for RCC with IVTT have been extensively reviewed in the previous literature, particularly in reference [7], there is a relative paucity of systematic summaries regarding robotic-assisted surgery. This gap in the literature highlights the need for a focused review on robotic techniques, particularly in the context of high- and low-level IVTT.

Our goal is to provide a comprehensive experience with laparoscopic (LAP) and robotic-assisted (RA) minimally invasive surgical techniques in patients with locally advanced RCC with IVTT. Meanwhile, we also performed a systematic review to compare the safety and feasibility of RA as regards the perioperative and oncological outcomes between RA and LAP, focusing on high- and low-level IVTT, respectively.

## 2. Methods

### 2.1. Search Strategy

To identify relevant studies for this systematic review, we performed a comprehensive search across multiple electronic databases, including MEDLINE (via PubMed), EMBASE, the Cochrane Central Register of Controlled Trials (CENTRAL), and Web of Science (WoS). The initial search was conducted in 2020, with regular updates performed to include the latest studies up to 2024. The search strategy employed both controlled vocabulary (e.g., MeSH terms in PubMed) and free-text terms to account for variations in terminology. Boolean operators (AND, OR) were used to combine the search terms, ensuring comprehensive coverage. The key search concepts included: “renal cell carcinoma”, “kidney neoplasm”, “intravenous tumor thrombus”, “thrombosis”, “laparoscopy”, “hand-assisted laparoscopy”, and “robotic surgical procedures” (Supplementary Text File). The database analysis was conducted on studies published from 2000 to 2024.

### 2.2. Inclusion Criteria

Study eligibility was assessed using a pre-specified framework based on population (P), intervention (I), comparator (C), outcome (O), and study design (S) (PICOS). Studies that did not sufficiently report these PICOS criteria were excluded from the review. The eligible study designs included retrospective or prospective case series and cohort studies. Case reports were excluded due to concerns over potential publication bias. Additionally, very small case series that included only 2–3 patients were also excluded, in order to enhance the methodological robustness and reduce potential selection bias.

### 2.3. Systematic Review Process

**Figure 1:**
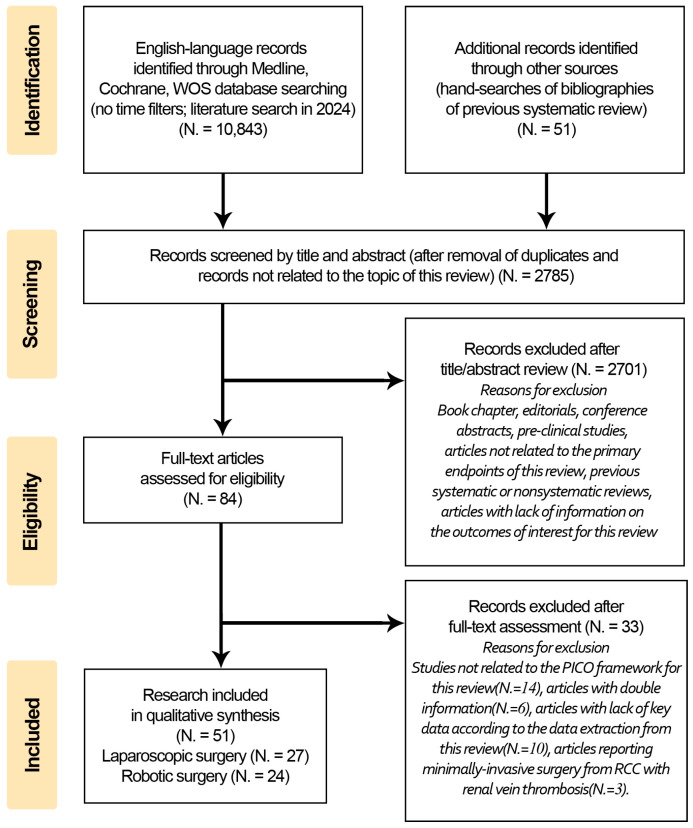
Flowchart illustrating the systematic literature search and study selection process for the review. The process followed the PRISMA guidelines and included four main stages: Identification: A total of 10,894 records (10,843 from database searches and 51 from additional sources) were identified; Screening: after removing duplicates, 2784 records were screened based on titles and abstracts, resulting in 84 articles for full-text assessment; Eligibility: of the 84 articles, 51 met the predefined PICOS criteria and were selected for qualitative analysis; and Included: Ultimately, 51 studies (39 case series and 12 cohort studies) were included in the systematic review. The flowchart provides a detailed visual representation of the study selection process, highlighting the exclusion criteria and the final number of studies included for analysis.

The systematic review followed the recommendations of the Preferred Reporting Items for the Systematic Reviews and Meta-Analyses (PRISMA) statement [8,9]. All identified studies (N = 10,843) were imported into EndNote (Clarivate, PA, USA) for screening and de-duplication (N = 2789). Two reviewers (Y.W., S.F.) independently screened the titles and abstracts of 2789 records, with disagreements resolved by a third reviewer (P.F.), who oversaw the review process. After excluding book chapters, editorials, conference abstracts, preclinical studies, studies on cadaveric models, previous reviews, and articles unrelated to the primary endpoints, 84 articles were assessed for eligibility. Of these, 54 studies that met all PICOS criteria were selected for qualitative analysis. The PRISMA flowchart illustrating the review process is shown in Figure 1.

Data were independently extracted by two authors (Y.W. and S.F.) using a pre-developed extraction form encompassing all elements of the PICOS framework. Risk of bias (ROB) assessments for cohort studies and case series were conducted independently by the same authors, following the Newcastle–Ottawa Scale (NOS) [10] and the Institute of Health Economics Delphi Tool (IHE–Delphi Tool) (Supplementary Tables S1–S5). Disagreements were resolved by a third reviewer (P.F.). The overall quality of the evidence was evaluated according to the Grading of Recommendations, Assessment, Development, and Evaluation (GRADE) guidelines [11] by Y.W. and S.F., with discrepancies adjudicated by P.F. (Supplementary Tables S6–S8). A narrative synthesis was employed for the qualitative data.

### 2.4. Classification of IVTT

**Figure 2:**
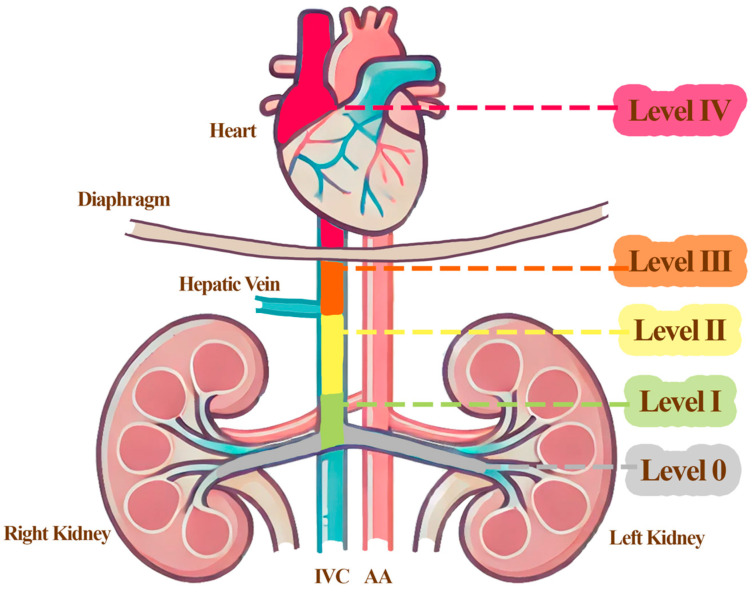
Schematic illustration of renal cell carcinoma (RCC) with intravenous tumor thrombus (IVTT) classification. Schematic representation of the Mayo classification for renal cell carcinoma (RCC) with intravenous tumor thrombus (IVTT). The illustration depicts the anatomical levels of tumor thrombus extension within the venous system as follows: Level 0: thrombus limited to the renal vein; Level I: thrombus extending into the inferior vena cava (IVC) but ≤ 2 cm above the renal vein ostium; Level II: thrombus extending into the IVC > 2 cm above the renal vein ostium but below the hepatic veins; Level III: thrombus extending into the IVC above the hepatic veins but below the diaphragm; Level IV: thrombus extending into the IVC above the diaphragm or into the right atrium.

To ensure consistency in the comparison of perioperative outcomes across studies, the level of intravenous cava tumor thrombus (IVTT) was classified based on a modified version of the Mayo Clinic grading system, as illustrated in Figure 2. In this classification, tumor thrombi were stratified into five levels according to their extent of cranial progression within the venous system:

- Level 0: tumor thrombus confined to the renal vein without extension into the inferior vena cava (IVC);
- Level I: extension into the IVC less than 2 cm above the renal vein;
- Level II: extension more than 2 cm above the renal vein but below the hepatic veins;
- Level III: extension to the intrahepatic IVC, up to but not beyond the diaphragm;
- Level IV: extension above the diaphragm or into the right atrium.

For this review, Levels 0–II were collectively defined as Low-Level IVTT, while Levels III–IV were categorized as High-Level IVTT, to facilitate the structured comparison of surgical strategies and perioperative indicators.

### 2.5. Statistical Analysis

Due to the absence of fully controlled studies, we combined the effect sizes of various outcomes into high-grade and low-grade categories to compare the efficacy of the two methods. Studies with missing values were excluded. For continuous variables, both fixed-effect and random-effect models were applied, with the variance components for random effects estimated using restricted maximum likelihood (REML). For dichotomous variables, single-group proportion meta-analysis was performed to pool event proportions (e.g., complication rates) across multiple studies. Heterogeneity between studies was evaluated using the I^2^ and Q statistics. All statistical analyses and forest plots were generated using R (version 4.3.1) and RStudio (version 2023.09.0+463).

## 3. Results

### 3.1. Characteristics of Included Studies

The key characteristics of the included studies in the review are shown in Table 1 and Table 2.

In total, 51studies were included, either as case series (N. = 39) or retrospective cohort studies (N = 12). No case reports were included in the review due to potential publication bias and the low quality of the evidence.

A total of 27 studies including 628 patients who underwent laparoscopic radical nephrectomy and IVTT thrombectomy were included in the LAP group as per Table 1. Most of them were case series (N = 20). Pure laparoscopic surgeries were performed in most studies, and only a few studies (N = 7) involved hand-assisted laparoscopic techniques. The number of patients included in the studies ranged from 3 to 88. Most of the included patients had a right-sided renal tumor (64.3%, 404/628). Most patients had a low-level tumor thrombus (95.9%, 602/628), and there were 244, 170, and 188 patients with Level 0, 1, and 2 tumor thrombi, respectively. The histology was clear cell RCC (ccRCC) in most cases, and papillary renal carcinoma (pRCC) comprised the second most common histology. T3b was the most common tumor pathological stage in the included studies.

A total of 24 studies including 671 patients who underwent robotic-assisted radical nephrectomy and IVTT thrombectomy were included in the RA group as per Table 2. Most of them were case series (N = 19). The number of patients included in the studies ranged from 2 to 120. Patients with a right-sided renal tumor (72.3%, 485/671) prevailed in them. Most patients had a low-level tumor thrombus (83.5%, 560/671), and patients with Level 2, 1, and 3 tumor thrombi ranked in the top three among them (58.5%/13.2%/12.5%, 394/89/84). The histology was ccRCC in most cases, and pRCC also ranked as the second most common histology, while the histopathological analysis was not reported in eight studies. pT3b was the main pathological stage in most studies, whereas more advanced stages, like pT3c or pT4, were also reported in 19 of them.

Overall, a hand-assisted laparoscopic approach was used in seven studies in the LAP group. In the LAP studies, a transperitoneal approach was performed in most series whilst a retroperitoneal approach was also reported in 12 of the latest studies, of which two studies revealed combined approaches for complex cases. In the RA studies, transperitoneal approach was performed in most, and one study reported a combined approach.

Most studies in the LAP series focused on the management of a right-sided tumor with low-level IVTT, especially Level 0. A pure laparoscopic technique performed in high-level IVTT was reported in only seven studies, and, notably, three studies included left-sided tumors with IVTT. Robotic-assisted management performed in patients with high-level IVTT was reported in 14 studies, and 4 studies tried to extend the indication to Level IV thrombi. Twenty-one studies in the RA group included left-sided tumors with IVTT. 

### 3.2. Comparison of RA and LAP Across Different Levels of IVTT

In accordance with the levels of IVTT involved, the studies in LAP and RA were categorized into High Level (Level 0–II thrombi) and Low Level (Level III–IV thrombi) groups, respectively. The perioperative outcomes we extracted for combination include estimated blood loss (EBL), operative time (OR time), transfusion rate, conversion, length of stay (LOS), perioperative complications, cancer specific mortality (CSM), distant metastasis, and local recurrence. In the LAP group, 8 eligible studies were categorized in the High Level, and 21 into the Low Level, with the aforementioned data extracted for pooled analysis. In two of the studies, data were repeatedly classified into two separate categories because they included two distinct cohorts, each of which could be independently assigned to a different category. Similarly, in the RA group, 14 and 8 eligible studies were categorized into the High Level and Low Level groups, respectively, and the same data were extracted. It is worth noting that not all eligible studies were applicable or clearly reported all perioperative outcomes. Thus, the number of eligible studies may vary across different pooled analyses.

#### 3.2.1. Operative Time

**Figure 3:**
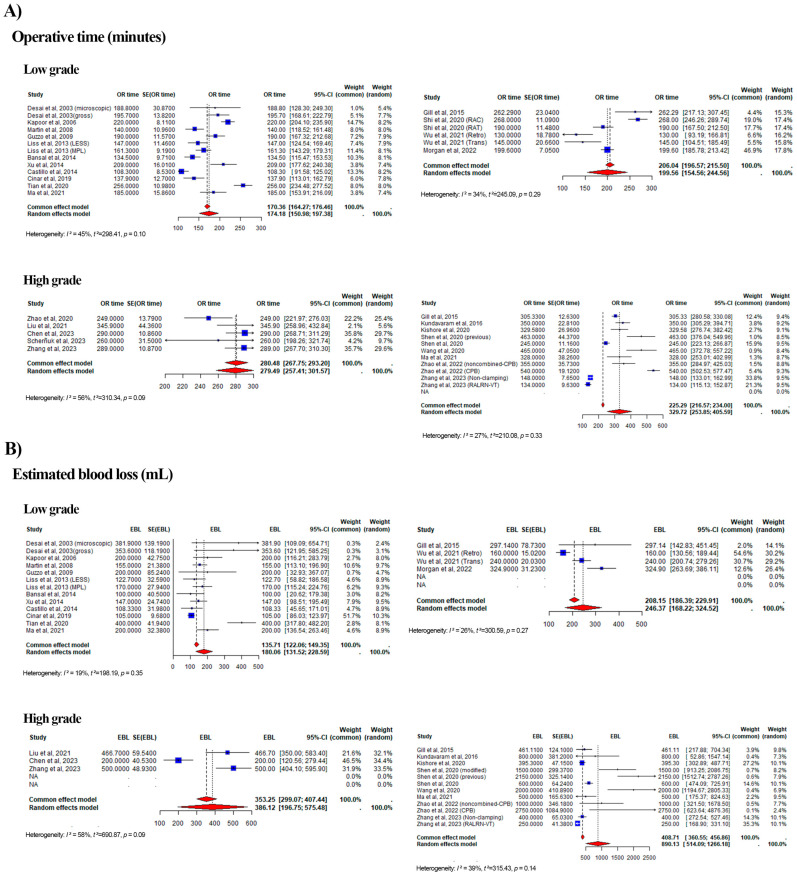
Forest plots summarizing the pooled effect sizes for perioperative outcomes, stratified [12,13,14,15,16,17,18,19,20,21,22,23,24,25,26,27,28,29,30,31,32,33,34,35,36,37,38,39,40,41,42,43,44,45,46,47,48,49,50,51,52,53,54,55,56,57,58,59,60,61]. (**A**) Forest plot for operative time, comparing laparoscopic (LAP) and robotic-assisted (RA) approaches. The plot displays median operative times (in minutes) with 95% confidence intervals for low-grade and high-grade tumor thrombi. (**B**) Forest plot for estimated blood loss (EBL), comparing LAP and RA approaches. The plot shows median EBL (in milliliters) with 95% confidence intervals for low-grade and high-grade tumor thrombi. Each plot includes heterogeneity statistics (I^2^) to assess variability across studies.

Of the 30 eligible studies, 10 and 4 studies from the RA group were categorized as High Level and Low Level, respectively, while 5 and 11 studies from the LAP group were categorized as High Level and Low Level, respectively (Figure 3A). The combined median operative time was 170.36 min (range: 164.27–176.46 min; weight range: 1–14.5%; I^2^ = 45%) for the Low Level LAP group compared to 206.04 min (range: 196.57–215.50 min; weight range: 4.4–46.9%; I^2^ = 34%) for the RA group, based on a common effect model. The combined median operative time was 280.48 vs. 225.29 min for the High Level for LAP vs. RA (range: 249.00–345.90 vs. 134.00–540.00 min; weight range: 2.1–35.8% vs. 0.9–33.8%; I^2^ = 56% vs. 27%).

#### 3.2.2. Estimated Blood Loss

Of the 28 eligible studies, 10 studies were categorized as High Level and 4 as Low Level in the RA group, while in the LAP group, 3 studies were categorized as High and 11 as Low Level (Figure 3B). The combined median EBL was 135.71 vs. 208.15 mL for the Low Level for LAP vs. RA based on the common effect model (range: 122.06–149.35 vs. 186.39–229.91 mL; weight range: 0.3–51.7% vs. 2–54.6%; I^2^ = 19% vs. 26%), while for the High Level, the combined median EBL was 353.25 vs. 408.71 mL for LAP vs. RA (range: 200.00–500.00 vs. 250.00–2750.00 mL; weight range: 21.6–46.5% vs. 0.1–35.3%; I^2^ = 58% vs. 39%).

#### 3.2.3. Transfusion Rate

**Figure 4:**
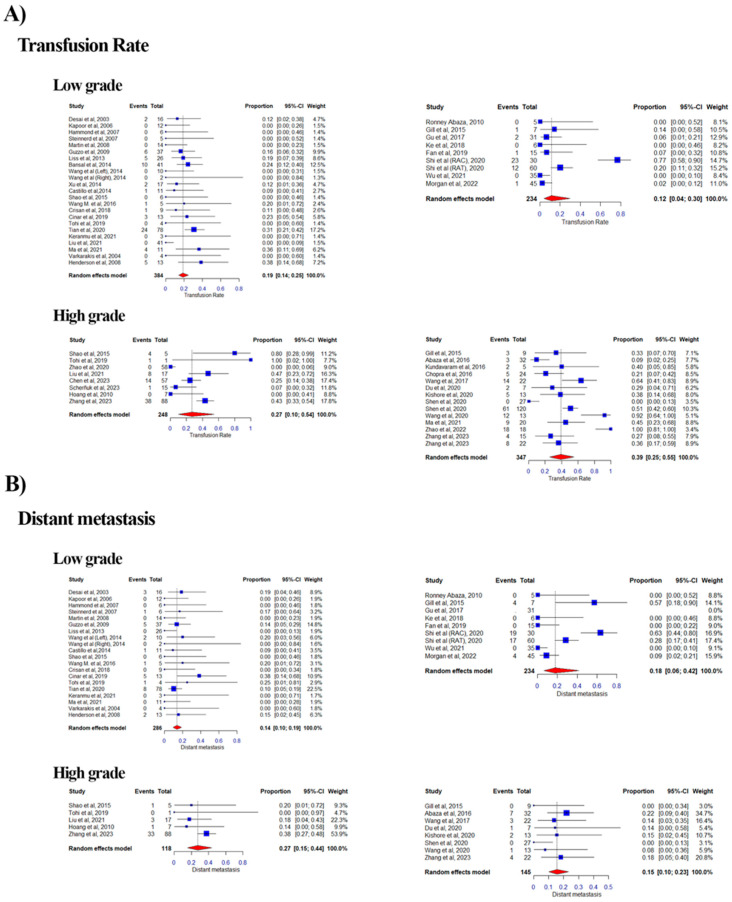
Forest plots of pooled effect sizes for transfusion rates and distant metastasis stratified by tumor thrombus level [12,13,14,15,16,17,18,19,20,21,22,23,24,25,26,27,28,29,30,31,32,33,34,35,36,37,38,39,40,41,42,43,44,45,46,47,48,49,50,51,52,53,54,55,56,57,58,59,60,61]. Forest plots summarizing the pooled effect sizes for transfusion rates and distant metastasis, stratified by low-grade (Mayo Levels 0-II) and high-grade (Mayo Levels III–IV) tumor thrombi. (**A**) Forest plot for transfusion rates, comparing laparoscopic (LAP) and robotic-assisted (RA) approaches. The plot displays the proportion of patients requiring blood transfusions with 95% confidence intervals for low-grade and high-grade tumor thrombi. (**B**) Forest plot for distant metastasis rates, comparing LAP and RA approaches. The plot shows the proportion of patients developing distant metastasis with 95% confidence intervals for low-grade and high-grade tumor thrombi. Each plot includes heterogeneity statistics (I^2^) to assess variability across studies.

Of the 51 eligible studies, 14 studies were categorized as High Level and 8 as Low Level in the RA group, while in the LAP group, 8 studies were categorized as High and 23 as Low Level. Notably, two studies from the LAP group were classified in both categories with distinct cohorts (Figure 4A). The combined proportion of perioperative transfusion of blood was 0.19 vs. 0.12 for the Low Level for LAP vs. RA based on a random effects model (range: 0.00–0.38 vs. 0.00–0.77; weight range: 1.3–17.2% vs. 8.1–15.2%; I^2^ = 0.1% vs. 0.84%). For the High Level, it was 0.27 vs. 0.39 for LAP vs. RA (range: 0.00–1.00 vs. 0.00–1.00; weight range: 7.7–17.8% vs. 3.4–10.3%; I^2^ = 0.72% vs. 0.72%).

#### 3.2.4. Conversion Rate

**Figure 5:**
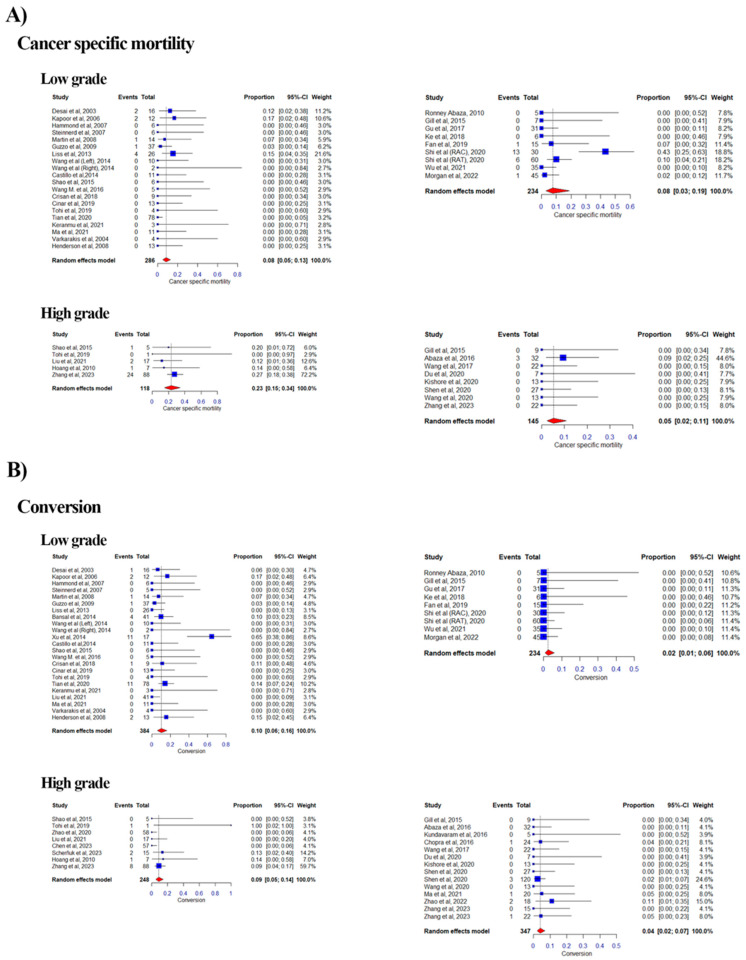
Forest plots of pooled effect sizes for oncological and surgical outcomes stratified by tumor thrombus level. [12,13,14,15,16,17,18,19,20,21,22,23,24,25,26,27,28,29,30,31,32,33,34,35,36,37,38,39,40,41,42,43,44,45,46,47,48,49,50,51,52,53,54,55,56,57,58,59,60,61] Forest plots summarizing the pooled effect sizes for oncological and surgical outcomes, stratified by low-grade (Mayo Levels 0–II) and high-grade (Mayo Levels III–IV) tumor thrombi. (**A**) Forest plot for cancer-specific mortality (CSM), comparing laparoscopic (LAP) and robotic-assisted (RA) approaches. The plot displays the proportion of cancer-specific mortality with 95% confidence intervals for low-grade and high-grade tumor thrombi. (**B**) Forest plot for conversion rates, comparing LAP and RA approaches. The plot shows the proportion of cases converted to open surgery with 95% confidence intervals for low-grade and high-grade tumor thrombi. Each plot includes heterogeneity statistics (I^2^) to assess variability across studies.

Of the 51 eligible studies, 14 studies were categorized as High Level and 8 as Low Level in the RA group, while in the LAP group, 8 studies were categorized as High and 23 as Low Level, with duplication due to the aforementioned reason (Figure 5B). The combined proportion of conversion to open surgery was 0.10 vs. 0.02 for the Low Level for LAP vs. RA based on a random effects model (range: 0.00–0.65 vs. 0.00–0.00; weight range: 2.8–10.2% vs. 10.6–11.4%; I^2^ = 0.42% vs. 0.00%). For the High Level, it was 0.09 vs. 0.04 for LAP vs. RA (range: 0.00–1.00 vs. 0.00–0.11; weight range: 3.1–59.7% vs. 3.9–24.6%; I^2^ = 0.4% vs. 0.0%).

#### 3.2.5. Length of Stay

**Figure 6:**
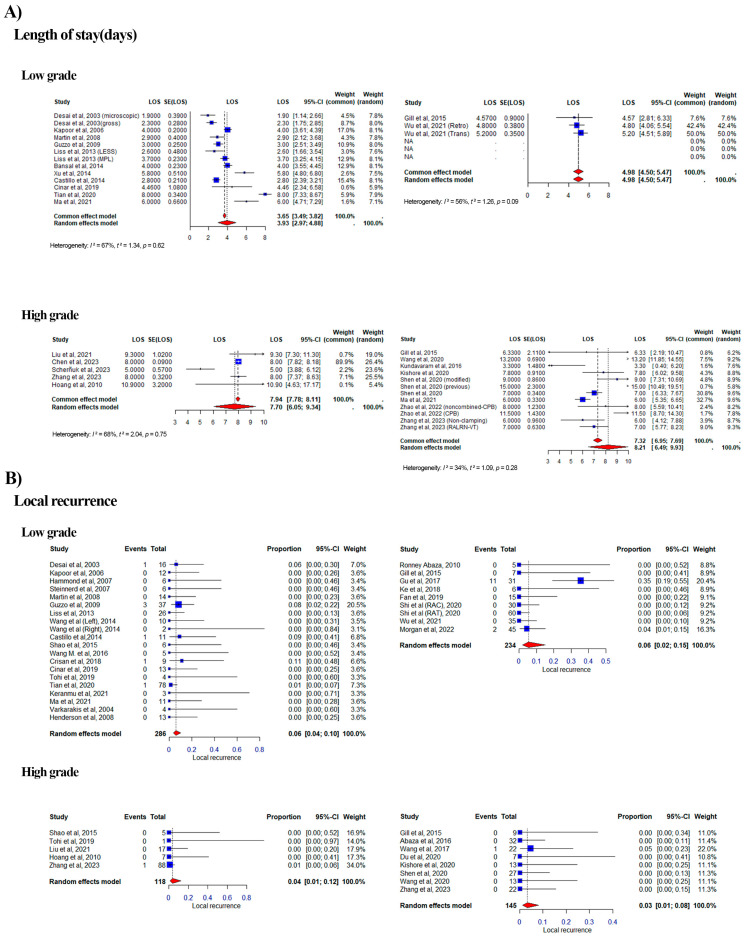
Forest plots of pooled effect sizes for length of stay and local recurrence stratified by tumor thrombus level. Forest plots summarizing the pooled effect sizes for length of stay (LOS) and local recurrence rates, stratified by low-grade (Mayo Levels 0–II) and high-grade (Mayo Levels III–IV) tumor thrombi [12,13,14,15,16,17,18,19,20,21,22,23,24,25,26,27,28,29,30,31,32,33,34,35,36,37,38,39,40,41,42,43,44,45,46,47,48,49,50,51,52,53,54,55,56,57,58,59,60,61]. (**A**) Forest plot for length of stay (LOS), comparing laparoscopic (LAP) and robotic-assisted (RA) approaches. The plot displays the median length of hospital stay (in days) with 95% confidence intervals for low-grade and high-grade tumor thrombi. (**B**) Forest plot for local recurrence rates, comparing LAP and RA approaches. The plot shows the proportion of patients experiencing local recurrence with 95% confidence intervals for low-grade and high-grade tumor thrombi. Each plot includes heterogeneity statistics (I^2^) to assess variability across studies.

Of the 28 eligible studies, 10 studies were categorized as High Level and 2 as Low Level in the RA group, while in the LAP group, 5 studies were categorized as High and 11 as Low Level (Figure 6A). The combined median LOS was 3.65 vs. 4.98 days for the Low Level for LAP vs. RA based on a common effect model (range: 3.49–3.82 vs. 4.50–5.47; weight range: 1.6–17.0% vs. 7.6–50.0%; I^2^ = 67 vs. 56%). For the High Level, it was 7.94 vs. 7.32 for LAP vs. RA (range: 5.00–10.90 vs. 3.30–15.00; weight range: 0.1–89.9% vs. 0.7–32.7%; I^2^ =68% vs. 34%).

#### 3.2.6. Minor Perioperative Complications Rate (Clavien-Dindo Grade I and II)

**Figure 7:**
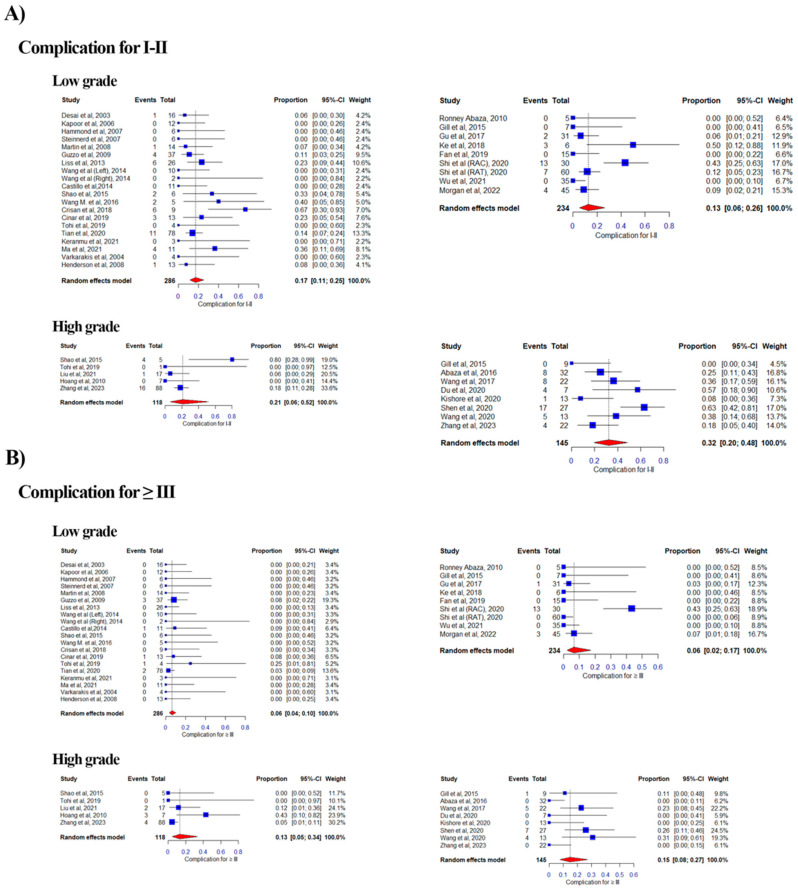
Forest plots of pooled effect sizes for perioperative complications stratified by tumor thrombus level [12,13,14,15,16,17,18,19,20,21,22,23,24,25,26,27,28,29,30,31,32,33,34,35,36,37,38,39,40,41,42,43,44,45,46,47,48,49,50,51,52,53,54,55,56,57,58,59,60,61]. (**A)** Forest plot for Clavien-Dindo Grade I–II (minor) complications, comparing laparoscopic (LAP) and robotic-assisted (RA) approaches. The plot displays the proportion of minor complications with 95% confidence intervals for low-grade and high-grade tumor thrombi. (**B**) Forest plot for Clavien-Dindo Grade ≥ III (major) complications, comparing LAP and RA approaches. The plot shows the proportion of major complications with 95% confidence intervals for low-grade and high-grade tumor thrombi. Each plot includes heterogeneity statistics (I^2^) to assess variability across studies.

Of the 39 eligible studies, 8 studies were categorized as High Level and 8 as Low Level in the RA group, while in the LAP group, 5 studies were categorized as High and 20 as Low Level, with duplication due to the aforementioned reason (Figure 7A). The combined proportion of minor perioperative complications was 0.17 vs. 0.13 for the Low Level for LAP vs. RA based on a random effects model (range: 0.00–0.67 vs. 0.00–0.50; weight range: 2.2–13.3% vs. 6.4–17.0%; I^2^ = 0.26% vs. 0.71%). For the High Level, it was 0.21 vs. 0.32 for LAP vs. RA (range: 0.00–0.80 vs. 0.00–0.63; weight range: 12.5–33.6% vs. 4.5–17.0%; I^2^ = 0.55% vs. 0.64%).

#### 3.2.7. Major Perioperative Complications Rate (Clavien-Dindo Grade ≥ III)

Of the 39 eligible studies, 8 studies were categorized as High Level and 8 as Low Level in the RA group, while in the LAP group, 5 studies were categorized as High and 20 as Low Level, with duplication due to the aforementioned reason (Figure 6B). The combined proportion of major perioperative complications was 0.06 vs. 0.06 for the Low Level for LAP vs. RA based on a random effects model (range: 0.00–0.25 vs. 0.00–0.43; weight range: 2.9–19.3% vs. 8.5–18.9%; I^2^ = 0.26% vs. 0.74%). For the High Level, it was 0.13 vs. 0.15 for LAP vs. RA (range: 0.00–0.43 vs. 0.00–0.31; weight range: 10.1–30.2% vs. 6.1–24.50%; I^2^ = 0.58% vs. 0.38%).

#### 3.2.8. Cancer Specific Mortality

Of the 39 eligible studies, 8 studies were categorized as High Level and 8 as Low Level in the RA group, while in the LAP group, 5 studies were categorized as High and 20 as Low Level, with duplication due to the aforementioned reason (Figure 5A). The combined proportion of CSM was 0.08 vs. 0.08 for the Low Level for LAP vs. RA based on a random effects model (range: 0.00–0.17 vs. 0.00–0.43; weight range: 2.7–21.6% vs. 7.8–18.8%; I^2^ = 0% vs. 0.72%). For the High Level, it was 0.23 vs. 0.05 for LAP vs. RA (range: 0.00–0.27 vs. 0.00–0.09; weight range: 2.9–72.2% vs. 7.7–44.6%; I^2^ = 0% vs. 0%).

#### 3.2.9. Distant Metastasis and Local Recurrence

Of the 41 eligible studies, 8 studies were categorized as High Level and 8 as Low Level in the RA group, while in the LAP group, 5 studies were categorized as High and 20 as Low Level, with duplication due to the aforementioned reason (Figure 7B). The combined proportion of distant metastasis was 0.14 vs. 0.18 for the Low Level for LAP vs. RA based on a random effects model (range: 0.00–0.38 vs. 0.00–0.63; weight range: 1.6–22.5% vs. 0.0–17.4%; I^2^ = 0% vs. 0.8%). For the High Level, it was 0.27 vs. 0.15 for LAP vs. RA (range: 0.00–0.38 vs. 0.00–0.22; weight range: 4.7–53.9% vs. 3.0–34.7%; I^2^ = 0% vs. 0%).

Of the 41 eligible studies, 8 studies were categorized as High Level and 8 as Low Level in the RA group, while in the LAP group, 5 studies were categorized as High and 20 as Low Level, with duplication due to the aforementioned reason. (Figure 6B) The combined proportion of local recurrence was 0.06 vs. 0.06 for the Low Level for LAP vs. RA based on a random effects model (range: 0.00–0.11 vs. 0.00–0.35; weight range: 3.1–20.5% vs. 8.8–20.4%; I^2^ = 0% vs. 0.69%). For the High Level, it was 0.04 vs. 0.03 for LAP vs. RA (range: 0.00–0.01 vs. 0.00–0.05; weight range: 14.0–34.0% vs. 11.0–22.0%; I^2^ = 0% vs. 0%).

Zhang et al. [61] conducted a comparative analysis of perioperative outcomes between 22 patients undergoing RA management and 148 patients undergoing LAP management, using a propensity-matched approach. Without stratification by IVTT level, they observed that the patients managed with RA had significantly shorter operative times (median 134 min vs. 289 min, *p* < 0.001), less estimated blood loss (median 250 mL vs. 500 mL, *p* < 0.001), and a reduced rate of perioperative transfusion (36.4% vs. 43.2%, *p* < 0.001). However, no significant differences were found between the groups regarding perioperative complications or postoperative length of stay.

### 3.3. Techniques for Laparoscopic Management

#### 3.3.1. Experience with Laparoscopy for Level 0–II Thrombi

The use of laparoscopy for managing Level 0–II inferior vena cava tumor thrombi (IVTT) has evolved significantly since the first hand-assisted laparoscopic surgery for right-sided renal cell carcinoma (RCC) with level I IVTT was reported by Sundaram et al. in 2002 [62]. Desai et al. [12] introduced the “thrombus milking” technique using an endoscopic stapler to remove intraluminal thrombi, while Varkarakis et al. [36] demonstrated the feasibility of hand-assisted laparoscopic surgery with Satinsky clamps for en bloc thrombus removal. Kapoor et al. [13] utilized intraoperative ultrasound to define thrombus margins, addressing the limited tactile feedback of laparoscopy. Early renal artery ligation, as described by Martin et al. [16], facilitated thrombus retraction and control in both pure and hand-assisted techniques. Guzzo et al. [17] emphasized the use of DeBakey graspers or Satinsky clamps to ensure thrombus-free renal vein transection.

Liss et al. [18] pioneered laparoendoscopic single-site surgery for selected patients, while Bansal et al. highlighted the benefits of early renal artery ligation in reducing tumor vascularity and thrombus retraction. Castillo et al. [22] employed a GelPort device for enhanced tactile feedback in complex cases. Wang et al. [20] introduced the pure retroperitoneal approach for left-sided RCC, enabling early renal hilar control and minimizing thrombus contact. For incomplete thrombus milking, Wang et al. [63] used partial IVC clamping and cold laparoscopic scissors for en bloc excision, preserving over 50% of the IVC lumen. Shao et al. [23] and Wang et al. further refined techniques with bulldog clamps and modified Rummel tourniquets for precise IVC control and partial wall resection.

Crisan et al. [25] combined retroperitoneal and transperitoneal approaches for complex cases, while Cinar et al. [26] used suction irrigation cannulas for thrombus milking. Tohi et al. introduced an IVC semi-occlusion technique to minimize thrombus fragmentation risks. Tian et al. [64] reported successful outcomes in 58 patients using a pure retroperitoneal approach. Keranmu et al. [65] employed a transmesocolic approach for left-sided RCC, and Ma et al. [57] developed a modified vein clamping technique for Level I–II thrombi. Liu et al. [30] introduced the Delayed Occlusion of the Proximal Inferior Vena Cava (DOPI) technique, utilizing pneumoperitoneum pressure to delay IVC clamping. Chen et al. [33] recently described the Pure Retroperitoneal Laparoscopic Peritoneum Incision Technique (PREP-IT) for improved IVC access in right-sided RCC. The above summary is presented in Table 3.

#### 3.3.2. Experience with Laparoscopy for Level III–IV Thrombi

For high-level IVTT (III–IV), Hoang et al. pioneered hand-assisted laparoscopic nephrectomy and thrombectomy for Level III thrombi, using intraoperative ultrasonography and extensive liver mobilization with IVC clamping [23]. Shao et al. [23] documented the first thoracoscope-assisted open atriotomy under cardiopulmonary bypass for Level IV thrombi, involving femoral and jugular cannulation and intercostal port guidance. Tohi et al. [27] described liver rotation and the Pringle maneuver for Level III thrombi, while Liu et al. [30] applied the DOPI technique to avoid immediate IVC clamping. Chen et al. [33] extended the PREP-IT technique to Level III thrombi, and Scherñuk et al. [34] reported an adjustable IVC and hepatic vein control technique for cases without hepatic vein involvement.

These advancements highlight the growing role of laparoscopy and robotic-assisted techniques in managing IVTT, with innovations in vascular control, thrombus extraction, and minimally invasive approaches improving surgical outcomes. The above summary is presented in Table 3.

#### 3.3.3. Experience with Robot-Assisted Laparoscopy for Level 0–II Thrombi

Robot-assisted laparoscopy has become a significant advancement in managing Level 0–II IVTT. In 2010, Abaza [66] reported the first case series of robotic radical nephrectomy (RN) and IVC thrombectomy (IVTTx) for right-sided RCC with Level I-II thrombi, utilizing a modified Rummel tourniquet for IVC cross-clamping and a percutaneous Satinsky clamp for tangential control. The robotic fourth arm was employed to retract the kidney, reducing the thrombus length within the IVC. Gill et al. [40] introduced a “minimal-touch” technique for Level II thrombi, emphasizing a “midline-first, lateral-last” strategy to minimize peri-caval tissue manipulation. Wang et al. [41] described side-specific techniques for left- and right-sided RCC, with preoperative renal artery embolization for left-sided cases to facilitate exposure and reduce blood loss. For right-sided RCC, clamping the left renal vein preserved venous return, while left-sided cases required right renal artery and vein clamping for full IVC control.

Abaza et al. [42] further refined techniques using Satinsky clamps for low-level thrombi, while Kundavaram et al. [43] employed Fogarty balloon catheters for proximal IVC occlusion, avoiding liver mobilization. Gu et al. [46] managed IVC tributaries extensively in 31 cases, using Hem-o-lok clips and sutures for secure vascular control. Fan et al. [49] reported dual-positioning strategies for left-sided RCC with thrombi extending beyond the SMA, while Rose et al. [50] highlighted pure robotic RN and IVTTx in 24 cases. Du et al. [51] utilized 3D reconstruction for precise IVC resection in left-sided RCC with Level II thrombi, preserving major collaterals. Shi et al. [55] reported selective robotic cavectomy or thrombectomy based on IVC wall invasion, employing tailored vascular stapling techniques. Zhang et al. [61] introduced the cephalic IVC non-clamping technique, using increased pneumoperitoneum pressure to control blood flow and minimize thrombus dislodgement. The above summary is presented in Table 4.

#### 3.3.4. Experience with Robot-Assisted Laparoscopy for Level III–IV Thrombi

For high-level thrombi (III–IV), Gill et al. [40] pioneered an “IVC-first, kidney-last” strategy in nine cases, prioritizing thrombus extraction and IVC repair before renal manipulation. Kundavaram et al. [43] described intracaval balloon occlusion and robotic biologic patch cavoplasty for complex Level III thrombi, while Wang et al. [47] emphasized liver mobilization and short hepatic vein (SHV) ligation based on thrombus location relative to the first and second porta hepatis (FPH and SPH). Du et al. [51] and Wang et al. [56] extended these techniques to Level III and IV thrombi, with a cardiopulmonary bypass (CPB) for Level IV cases and thoracoscopy-assisted thrombectomy for intra-atrial thrombi. Shen et al. [53] introduced a modified sequential vascular control strategy to enable early CPB cessation, while Ma et al. [57] used rubber vascular bands for sequential IVC clamping. Zhao et al. [60] reported a stepwise thrombus-lowering technique, reducing CPB duration by transitioning vascular control from CPB to suprahepatic and retrohepatic IVC control.

These advancements demonstrate the efficacy and versatility of robot-assisted laparoscopy in managing complex IVTT, with innovations in vascular control, thrombus extraction, and minimally invasive approaches improving surgical outcomes. The above summary is presented in Table 4.

### 3.4. LAP: Does Hand-Assisted Value Still Exist?

Kalra et al. [67] even suggested that hand-assisted management could be a viable option for complex cases like large renal masses or significant perirenal inflammation and demonstrated its feasibility as a bridge between pure laparoscopic and open surgery in the early stages.

Both Henderson et al. [37] and Martin et al. [16] emphasized that hand-assisted laparoscopic management provided superior tactile feedback compared to pure laparoscopic techniques as it facilitates the achievement of negative margins and better control of major hemorrhage without conversion to open surgery.

## 4. Discussion

Minimally invasive techniques, including laparoscopic and robot-assisted approaches, have become feasible for managing RCC with IVC tumor thrombi, offering superior perioperative outcomes and maintaining oncological safety [68]. Over the past two decades, these techniques have evolved, but variability in outcomes highlights the need for comparative evaluation [47]. This review synthesizes perioperative data and surgical innovations to assess the relative advantages and limitations of laparoscopic and robot-assisted techniques.

### 4.1. Perioperative Outcomes

*Operative Time:* Laparoscopic techniques showed shorter times for low-level thrombi, while robot-assisted approaches had shorter times for high-level cases.

*Blood Loss and Transfusion:* Minimal differences were observed for low-level thrombi, but robot-assisted techniques had higher transfusion rates for high-level cases, likely due to more Level IV thrombi requiring a cardiopulmonary bypass (CPB).

*Complications:* Robot-assisted techniques had lower minor complication rates for low-level thrombi but increased rates for high-level cases. Major complications were similar across both approaches.

*Oncological Outcomes:* Robot-assisted techniques showed stable cancer-specific mortality (CSM) and distant metastasis rates across thrombus levels, while laparoscopic techniques exhibited increased CSM and metastasis rates for high-level thrombi.

### 4.2. Technical Advancements

*Laparoscopy:* Hand-assisted techniques provided early tactile feedback, while innovations like DOPI IVC management and PREP-IT expanded capabilities for high-level thrombi. However, experience remains limited.

*Robot-Assisted Surgery:* This involved replicating laparoscopic techniques and robotic surgery introduced innovations like intracaval balloon occlusion and detailed IVC control. For high-level thrombi, it often requires liver mobilization, CPB, and multidisciplinary collaboration, and there is growing experience in complex cases like double-thrombi and IVC reconstruction.

### 4.3. Imaging and Expertise

Preoperative and intraoperative imaging, such as intraluminal ultrasound and 3D reconstruction, play a critical role in surgical planning and decision-making. It is important to note that, while our review excluded case reports to focus on studies with statistical significance, some case reports have demonstrated the successful application of advanced surgical techniques, including 3D reconstruction, in managing rare and complex cases [68]. However, most studies originate from highly experienced single-center teams, raising questions about broader applicability and underscoring the need for multicenter studies.

### 4.4. Patient Selection and Expanding Eligibility Criteria

In the early stages of laparoscopic surgery for RCC with venous tumor thrombus, the immaturity of surgical techniques significantly limited patient eligibility. Pure laparoscopic approaches were initially reserved for cases with small tumor volume and low-level, non-adherent thrombi. For patients with more advanced or complex thrombi—such as larger tumor burdens, bland thrombus extension, or vessel wall invasion—surgeons often relied on hand-assisted techniques to provide tactile feedback or planned early conversion to open surgery to ensure safety. These preparatory strategies contributed to the relatively high conversion rates observed in early laparoscopic series.

With the evolution of laparoscopic instrumentation and vascular control techniques —including innovations like the DOPI strategy and the PREP-IT technique that facilitates en bloc thrombus control within the IVC—the technical feasibility of pure laparoscopy has markedly improved. Although few studies explicitly redefine eligibility criteria for pure laparoscopy, increasing reports of the successful management of Level II–III thrombi, complex vascular reconstructions, and even IVC wall excision without hand assistance or conversion reflect a clear trend toward broader patient inclusion and lower conversion rates.

Robot-assisted laparoscopic surgery, inherently building upon conventional laparoscopic experience, has followed a similar trajectory. Typically performed by high-volume urologists with significant laparoscopic expertise, robotic surgery was initially limited to early-stage thrombi. However, the advent of refined vascular control techniques—such as the use of intracaval balloon occlusion, stepwise IVC clamping under robotic visualization, and the secure control of hepatic veins—has enabled the treatment of more challenging thrombi. Studies such as those by Nayak et al. and Bai et al. highlight the feasibility of robotic approaches in managing Level III–IV thrombi, even in cases involving double luminal thrombi or IVC reconstruction, with favorable perioperative outcomes and low conversion rates. These advances imply a substantial expansion in the generalizability of robotic-assisted approaches to a wider spectrum of patients.

### 4.5. Cost Effectiveness

The cost-effectiveness of robotic-assisted versus laparoscopic approaches remains a topic of debate. While RA offers advantages in terms of operative time and blood loss, particularly for high-level IVTT, the high upfront costs of robotic systems may limit their widespread adoption. Future studies should evaluate the long-term cost-effectiveness of these approaches in relation to patient outcomes.

Limitations: There was the exclusion of case reports to reduce bias, and the review was limited to English-language publications. Heterogeneity in data prevents direct comparisons, necessitating descriptive interpretation. Inconsistent reporting highlights the need for standardized surgical protocols. Long-term oncological outcomes, especially for robotic approaches, remain underreported.

## 5. Conclusions

The perioperative results and oncological outcomes of robotic surgery in all grades of IVTT were not inferior to those of laparoscopic surgery and were within an acceptable range. Robotic surgery is technically feasible for patients with RCC and IVC tumor thrombus and may be superior to laparoscopic surgery

However, most of these robotic surgery cases come from a few senior surgeons. Therefore, there is still a long way to go in the training and promotion of robotic surgery for RCC patients with IVC tumor thrombus in urology.

The evidence is currently insufficient to draw reliable conclusions about the long-term oncologic outcomes of this technique. Future research is needed to establish the non-inferiority of this strategy compared to laparoscopic surgery and to develop robust selection criteria as an initial step in assessing the reproducibility of robotic surgery beyond expert surgical teams.

## Figures and Tables

**Table 1:** Key characteristics of studies on laparoscopic radical nephrectomy with inferior vena cava thrombectomy.

Author/Year	Study Type	Approach	Number of Patients	Age (Median or Mean, yo)	Tumor Size (Median or Mean, cm)	RCC Histology	IVTT Level	Pathological Stage
Desai, 2003 [12]	case series	LAP	16	57.8	3.3 ± 6.5 (3–4)	ccRCC, pRCC, sRCC, gRCC	0: 16; invasive: 2	T3aN0M0:13 T4N0M0:3
Kapoor, 2006 [13]	case series	LAP/HLAP	12	62.3	8 (IQR = 2)		0:12	
Hammond, 2007 [14]	case series	LAP	6	55.8	9.5 (7.5–11.5)	ccRCC, Other RCC	0:06	T3aNxM0:4 T3aNxM0:2
Steinnerd, 2007 [15]	case series	LAP	5	59.8	5.5 (4.6–6.0)	ccRCC, pRCC	0: 5; invasive: 1	T3aN0M0:5
Martin, 2008 [16]	case series	LAP/HLAP	14	65	7.3 ± 2.2	ccRCC	0: 10; I: 3; II: 1	T3bNx
Guzzo, 2009 [17]	case series	LAP	37	65	6 (3.5–12)	ccRCC, pRCC, sRCC	0:37	T3aN0M0:32 T3aN1 + T3aNxM1:5
Liss, 2013 [18]	case series	LAP	26	60.8	7.9 ± 2.2	ccRCC, Other RCC	0:26	T3aNxM0
Bansal, 2014 [19]	case series	LAP	41	64.4	9.3 (4–22)	ccRCC, pRCC, Other RCC	0: 39; I: 3	T3aNxM0:34 T3aNxM1:5 T3bNxM1:2
Wang(left), 2014 [20]	case series	LAP	10	64	6.3 (5.0–8.5)	ccRCC(9), chRCC(1)	0:10	T3aN0M0:8 T3aN0M1:2
Xu, 2014 [21]	cohort study	LAP	17	50.1	7.9 ± 2.6		0: 5; I: 12	T3aNxM0:5 T3bNxM0:12
Castillo, 2014 [22]	case series	LAP/HLAP	11	66.8	10.5 ± 2.5	ccRCC(10), ccRCC + sRCC(1)	0:11	T3aN0M0
Shao, 2015 [23]	case series	LAP	11	53.5	7.8 (6.5–9.3)	ccRCC(10), pRCC(1)	II: 6; IV: 5	T3bN0M0:9 T3bN1M0:2
Wang M, 2016 [24]	case series	LAP	5	57	6.9 (3.5–9)	ccRCC	II: 5	T3bN0M0
Crisan, 2018 [25]	case series	LAP	9	61	7.6 (5.5–10.5)	ccRCC(8), sRCC(1)	0: 3; I: 3; II: 3	T3bN0M0:3 T3bN1M0:6
Cinar, 2019 [26]	case series	LAP	13	61.6	9.5 × 7.3 (5–14)	ccRCC(11), pRCC(2)	0/I:11; II:2; invasive: 4	T3bN0M0:6 T3bN1M0:2 T3bN0M1:5
Tohi, 2019 [27]	case series	LAP/HLAP	5	63	7.3 (3.5–11)	ccRCC	I: 1; II: 3; III:1	T3aN0M0:1 T3bN0M0:1 T3cN0M0:3
Tian, 2020 [28]	case series	LAP	78	59	8.3 (IQR: 6.9–9.5)	ccRCC(70), pRCC(7), chRCC(1)	0: 28; I: 27; II: 23	T3aNxM0 + T3aNxM1:28 T3bN0M0 + T3bNxM1:50
Zhao, 2020 [29]	cohort study	LAP	58	61.2	7.9 ± 2.3	ccRCC(52), Other RCC(6)	0: 22; I:23; II: 10; III: 3	T3a:22 T3b:33 T3c:3
Liu, 2021 [30]	cohort study	LAP	17	52.2	8.1 ± 3.4 (3.5–11)	ccRCC(12), pRCC(1), chRCC(1), other(3)	II: 13; III:4	T3bNxMx = 13; T3cNxMx = 4
Liu, 2021 [31]	cohort study	LAP	41	60.2	7.98 ± 2.16	ccRCC(37), other(4)	I: 26; II: 15	T3aN0M0 + T3aN1M0:32 T3bN0M1 + T3bN1M1:9
Ma, 2021 [32]	case series	LAP	11	57	7.20 (IQR: 6.00–10.50)	ccRCC	I: 6; II: 5	T3bN0M0 = 9; T3bN0M1 = 2
Chen, 2023 [33]	cohort study	LAP	57	61	8.0 (M; IQR = 6.1–9.9)	ccRCC(48), pRCC(4), chRCC(1), other(4)	I: 25; II: 29; III:3	T3bN0Mx = 56; T3bNxM0 = 45
Scherñuk, 2023 [34]	cohort study	LAP	15	61.9	9.00 (M; IQR = 6.50–11.90)	ccRCC(13), pRCC(1), other(1)	I: 7; II: 27; III: 6	T3bNxMx = 15; T3bN1Mx = 3; T3bNxM1 = 4
Zhang, 2023 [35]	cohort study	LAP	88	60	6.4 (M; IQR = 5.8–9.8)	ccRCC(78), pRCC(9), chRCC(1), other(6)	I: 20; II: 61; III: 7; invasive: 21	T3a = 15; T3b = 39; T3c = 36; T4 = 4; N1 = 54; M1 = 21
Varkarakis, 2004 [36]	case series	HLAP	4	56	9 (6–13)		II: 4	T3bNx
Henderson, 2008 [37]	case series	HLAP	13	68.8	8.1 (4.5–12)		0:13	T3aN0M0:12 T3aN1M0:1
Hoang, 2010 [38]	case series	HLAP	7	66	9.1 (5.7–12.8)		II: 6; III: 1	T3bNxM0:5 T3bNxM1:1 T3cNxM0:1

**Table 2:** Key characteristics of studies on robotic radical nephrectomy with inferior vena cava thrombectomy.

Author/Year	Study Type	approach	Number of Patients	Age (Median or Mean, yo)	Tumor Size (Median or Mean, cm)	RCC Histology	IVTT Level	Pathological Stage
Ronney Abaza, 2010 [39]	case series	ROB	5	64	10.4 (7.8–15.5)		I: 2, II: 3,	T3bN1M0:4 T3bN1M1:1
Gill, 2015 [40]	case series	ROB	16	66.2	9.7 (6.5–19.5)		II: 7, III: 9, invasive: 2	T3bN0M0: 28 T3bN1M0: 4 T3bN0M1: 4
Wang, 2015 [41]	case series	ROB	17	61	5.8 (4–10)		I: 4, II: 3	T3bN0M0:16 T3bN1M0:2 T3bN0M1:1
Abaza, 2016 [42]	case series	ROB	32	63	9.6 (5.4–20)		I/II: 30, III: 2	
Kundavaram, 2016 [43]	case series	ROB	5	59.3	8.0 (5.5–9.5)	ccRCC(3), pRCC(1)Collecting duct CA(1)	II: 1, III: 3, invasive: 1	T3cN1Mx:1 T3cN0Mx:2 T4N2M0 T3cN0Mx
Chopra, 2016 [44]	case series	ROB	24	64	8.5 (5.3–19.5)	ccRCC(23), pRCC(1)	II: 13, IV: 1	T3b:19 T3c:3 T4:3 TxN1Mx:3 TxNxM1:5
Davila, 2016 [45]	case series	ROB	10	55.6	1.9–11			
Gu, 2017 [46]	cohort study	ROB	31	55.7	7.3 (SD = 3.0)	ccRCC(26), pRCC(3), Other(2)	I: 10, II: 21	T3bN0Mx:29 T3bN1Mx:2
Wang, 2017 [47]	case series	ROB	22	58.5	7.8 (2.5–15.0)	ccRCC(16), pRCC(2), Other(4)	II: 20, III: 2, invasive: 3	T3bN0M0:17 T3bN0M1:4 T3cN0M1:1
Ke, 2018 [48]	case series	ROB	6	57	7.2 (3.2–8.4)	ccRCC(4), pRCC(1), Other(1)	0: 3, I: 1, II: 2,	T3aN0M0:3 T3bN0M0:1 T3bN1M0:1 T3bN0M1:1
Fan, 2019 [49]	case series	ROB	15	62	8.1(3–10)	ccRCC(4), pRCC(1), Collecting duct CA(1)	0:15	T3aN0M0:5 T3aN1Mx:1 T4NxM0:2 T3aNxM1:2 T3aN0M0:4 T3aN1M0:1
Rose, 2019 [50]	cohort study	ROB	24			ccRCC(20), Other(4)	I: 2, II: 22	T3bNxM0:19 T3bNxM1:5
Du, 2020 [51]	case series	ROB	7	58	9.2 (6.0–15.0)	ccRCC(5), pRCC(2)	II: 5, III: 2, invasive: 5	T3bN0M0:2 T3cN0M0:5
Kishore, 2020 [52]	case series	ROB	13	56.5	9.25	ccRCC(12), pRCC(1)	I: 5, II: 7, III: 1,	T3aN0M0:1 T3bN0M0:8 T3bN1M0:2 T3bN0M1:2
Shen, 2020 [53]	case series	ROB	27	60.3			III: 14, IV: 13	T3bNxMx:4 T3cNxMx:7 T4NxMx:1 T3bNxMx:5 T3cNxMx:8 T4NxMx:2
Shen, 2020 [54]	case series	ROB	120	54.1	7.9 (SD = 3.1)	ccRCC(81), pRCC(14), Other(25)	I: 30, II: 74, III: 14, IV: 2	T3b:93 T3c:23 T4:4 Nx:70 N0:35 N1:15 M0:107 M1:13
Shi, 2020 [55]	case series	ROB	90	54	8.6 (2.5–19.0)	ccRCC(77), pRCC(13)	II: 90, invasive: 18	T3b + T3c:14 T4:17 TxN1Mx:4 TxNxM1:6 T3b + T3c:59 T4:1 TxN1Mx:8 TxNxM1:6
Wang, 2020 [56]	case series	ROB	13	57.5	8.2 (SD = 4.3)	ccRCC	III: 7, IV: 6	T3bN0M0:4 T3bN1M0:1 T3cN0M0:7 T4N0M1:1
Ma, 2021 [57]	case series	ROB	20	59	67 cm^2^ (IQR: 40–91 cm2)	ccRCC(9), other(11)	0: 2, I: 3, II: 12, III: 3, invasive: 1	T3aNxM0:2; T3bNxM0:13; T3cNxM0:3; T4NxM0: 2
Wu, 2021 [58]	cohort study	ROB	35	58	6.9 (IQR:3.0–7.2)	ccRCC(28), other(7)	: 10, II: 25	T3bN0M0 = 14; T3bN1M0 = 2 T3bN0M0 = 15; T3bN1M0 = 3; T3bN0M1 = 1
Morgan, 2022 [59]	case series	ROB	45	64.9	4.3 (range = NA; SD = 1.3)	ccRCC(41); pRCC(2); other(2)	0:45	T3aN0M0 = 45
Zhao, 2022 [60]	cohort study	ROB	18	55.3	8.9 (range = NA; SD = 2.9)	ccRCC(15); pRCC(1); other(2)	III: 10, IV: 8	T3bNxMx = 10; T4NxMx = 1 or T3NxM1 = 1 T3cNxMx = 7
Zhang, 2023 [35]	case series	ROB	30	60	7.3 (M; range = NA; IQR = 6.1–8.7)	ccRCC(23), pRCC(3), other(4)	II: 28, III: 2, invasive: 13	T3bNxMx = 8; T3cNxMx = 7; T3N1Mx = 3; T3NxM1 = 4 T3bNxMx = 8; T3cNxMx = 7; T3N1Mx = 3; T3NxM1 = 7
Zhang, 2023 [61]	cohort study	ROB	22	58	6.5 (M; range = NA; IQR = 5.8–9.6)	ccRCC(21), pRCC(1)	I: 5, II: 15, III: 2, invasive: 4	T3a = 5; T3b = 10; T3c = 7; N1 = 13; M1 = 6

**Table 3:** Summary oflaparoscopy surgical techniques.

Category	Technique	Description
Laparoscopy for Level 0–II Thrombi	Hand-assisted Laparoscopic Surgery	First reported for right-sided RCC with level I IVTT using Satinsky clamps for en bloc thrombus removal
	Thrombus Milking Technique	Removal of intraluminal thrombi using an endoscopic stapler
	Intraoperative Ultrasound	Utilized to define thrombus margins, addressing limited tactile feedback
	Early Renal Artery Ligation	Facilitates thrombus retraction and control in both pure and hand-assisted techniques
	DeBakey Graspers/Satinsky Clamps	Ensures thrombus-free renal vein transection
	Laparoendoscopic Single-Site Surgery	Pioneered for selected patients
	Early Renal Artery Ligation	Reduces tumor vascularity and thrombus retraction
	GelPort Device	Enhances tactile feedback in complex cases
	Pure Retroperitoneal Approach	Introduced for left-sided RCC, enabling early renal hilar control
	Partial IVC Clamping	Used for incomplete thrombus milking, preserving over 50% of the IVC lumen
	Bulldog Clamps/Modified Rummel Tourniquets	Provides precise IVC control and partial wall resection
	Combined Retroperitoneal and Transperitoneal Approaches	Applied for complex cases
	Suction Irrigation Cannulas	Used for thrombus milking
	Modified Vein Clamping Technique	Developed for Level I-II thrombi
	Delayed Occlusion of the Proximal Inferior Vena Cava (DOPI)	Utilizes pneumoperitoneum pressure to delay IVC clamping
	Pure Retroperitoneal Laparoscopic Peritoneum Incision Technique (PREP-IT)	Improves IVC access in right-sided RCC
Laparoscopy for Level III-IV Thrombi	Hand-assisted Laparoscopic Nephrectomy and Thrombectomy	Pioneered for Level III thrombi using intraoperative ultrasonography and IVC clamping
	Thoracoscope-assisted Open Atriotomy	First reported for Level IV thrombi under cardiopulmonary bypass
	Liver Rotation and Pringle Maneuver	Described for Level III thrombi
	DOPI Technique	Applied to avoid immediate IVC clamping
	PREP-IT Technique	Extended to Level III thrombi
	Adjustable IVC and Hepatic Vein Control Technique	Developed for cases without hepatic vein involvement

**Table 4:** Summary of robot-assisted surgical techniques.

Category	Technique	Description
Robot-Assisted Laparoscopy for Level 0–II Thrombi	Robotic Radical Nephrectomy (RN) and IVC Thrombectomy (IVTTx).	First case series for right-sided RCC with Level I-II thrombi using modified Rummel tourniquet and percutaneous Satinsky clamp
	Minimal-Touch Technique	Emphasizes “midline-first, lateral-last” strategy for Level II thrombi to minimize peri-caval tissue manipulation
	Side-Specific Techniques	Preoperative renal artery embolization for left-sided RCC; left renal vein clamping for right-sided RCC
	Satinsky Clamps	Refined techniques for low-level thrombi
	Fogarty Balloon Catheters	Proximal IVC occlusion without liver mobilization
	IVC Tributaries Management	Hem-o-lok clips and sutures for secure vascular control
	Dual-Positioning Strategies	For left-sided RCC with thrombi extending beyond the SMA
	Pure Robotic RN and IVTTx	Reported in 24 cases
	3D Reconstruction	Precise IVC resection in left-sided RCC with Level II thrombi
	Selective Robotic Cavectomy/Thrombectomy	Based on IVC wall invasion, using tailored vascular stapling techniques
	Cephalic IVC Non-Clamping Technique	Increased pneumoperitoneum pressure to control blood flow
Robot-Assisted Laparoscopy for Level III-IV Thrombi	IVC-First, Kidney-Last Strategy	Prioritizes thrombus extraction and IVC repair before renal manipulation
	Intracaval Balloon Occlusion and Robotic Biologic Patch Cavoplasty	For complex Level III thrombi
	Liver Mobilization and SHV Ligation	Based on thrombus location relative to FPH and SPH
	Cardiopulmonary Bypass (CPB) and Thoracoscopy-Assisted Thrombectomy	For Level IV cases and intra-atrial thrombi
	Modified Sequential Vascular Control Strategy	Enables early CPB cessation
	Rubber Vascular Bands	Sequential IVC clamping
	Stepwise Thrombus-Lowering Technique	Reduces CPB duration by transitioning vascular control

## Supplementary Materials

The following supporting information can be downloaded at: https://www.mdpi.com/article/10.3390/curroncol32050256/s1, Table S1: Risk of bias assessment among LAP-related cohort studies included in the review evaluated by the Newcastle-Ottawa-Scale (NOS) for cohort studies; Table S2: Risk of bias assessment among ROB-related cohort studies included in the review evaluated by the Newcastle-Ottawa-Scale (NOS) for cohort studies. Table S3: Risk of bias assessment among LAP-related case-series reports included in the review evaluated by the IHE-Delphi-Tool for case-series studies. Table S4: Risk of bias assessment among HALP-related case-series reports included in the review evaluated by the IHE-Delphi-Tool for case-series studies. Table S5: Risk of bias assessment among ROB-related case-series reports included in the review evaluated by the IHE-Delphi-Tool for case-series studies. Table S6: Quality of evidence evaluation among LAP-related studies included in the review evaluated by the Grading of Recommendations, Assessment, Development, and Evaluation (GRADE) system. Table S7: Quality of evidence evaluation among HALP-related studies included in the review evaluated by the Grading of Recommendations, Assessment, Development, and Evaluation (GRADE) system. Table S8: Quality of evidence evaluation among ROB-related studies included in the review evaluated by the Grading of Recommendations, Assessment, Development, and Evaluation (GRADE) system.
