# Supplementary material for: Minimally Invasive Surgical Techniques for Renal Cell Carcinoma with Intravenous Tumor Thrombus: A Systematic Review of Laparoscopic and Robotic-Assisted Approaches
Source: Curr Oncol. 2025 Apr 28;32(5):256. doi: 10.3390/curroncol32050256 (PMC12109617; doi:10.3390/curroncol32050256)
Supplement: Supplementary file 1 [file curroncol-32-00256-s001.zip › curroncol-3545779-supplementary/Table for GRADE (Tables S6-S8).pdf]

Supplementary Table S6 – Quality of evidence evaluation among LAP-related studies included in the review evaluated by the Grading of Recommendations, Assessment, Development, and Evaluation (GRADE) system.

Study	Appraiser	Study Design	
		Randomized Trials (RT)	Observational Studies (OS)
Desai et al. 2003	S. F.		√
	Y. W.		√
Kapoor et al. 2006	S. F.		√
	Y. W.		√
Hammond et al. 2007	S. F.		√
	Y. W.		√
Steinnerd et al. 2007	S. F.		√
	Y. W.		√
Martin et al. 2008	S. F.		√
	Y. W.		√
Guzzo et al. 2009	S. F.		√
	Y. W.		√
Liss et al. 2013	S. F.		√
	Y. W.		√
Bansal et al. 2014	S. F.		√
	Y. W.		√
Wang et al (Left). 2014	S. F.		√
	Y. W.		√
Wang et al (Right). 2014	S. F.		√
	Y. W.		√
Xu et al. 2014	S. F.		√
	Y. W.		√
Castillo et al. 2014	S. F.		√
	Y. W.		√
Shao et al. 2015	S. F.		√
	Y. W.		√
Wang et al. 2016	S. F.		√
	Y. W.		√

Lower if			Initial Quality	
Indirectness (III)	Inconsistency （II）	Risk of Bias (I)	Low	High
0	0	0	√	
0	0	0	√	
0	0	0	√	
0	0	0	√	
0	0	0	√	
0	0	0	√	
0	0	0	√	
0	0	0	√	
0	0	0	√	
0	0	0	√	
0	0	0	√	
0	0	0	√	
0	0	0	√	
0	0	0	√	
0	0	0	√	
0	0	0	√	
0	0	0	√	
0	0	0	√	
0	0	0	√	
0	0	0	√	
0	0	-1	√	
0	0	-1	√	
0	0	0	√	
0	0	0	√	
0	0	0	√	
0	0	0	√	
0	0	0	√	
0	0	0	√	

Higher if			
Dose Response (II)	Large Effect (I)	Publication Bias (V)	Imprecision (IV)
0	0	-1	0
0	0	-1	0
0	0	-1	0
0	0	-1	0
0	0	-1	0
0	0	-1	0
0	0	-1	0
0	0	-1	0
0	0	-1	0
0	0	-1	0
0	0	-1	0
0	0	-1	0
0	0	-1	0
0	0	-1	0
0	0	-1	0
0	0	-1	0
0	0	-1	0
0	0	-1	0
0	0	-1	0
0	0	-1	0
0	0	-1	0
0	0	-1	0
0	0	-1	0
0	0	-1	0
0	0	-1	0
0	0	-1	0
0	0	-1	0
0	0	-1	0

Disagreement Adjudication by P. F.	With/Without Disagreement	All Plausible Residual Confounding (III)
\	Without	+1
		+1
\	Without	+1
		+1
S. F.	With	+1
		+2
\	Without	+1
		+1
\	Without	+1
		+1
\	Without	+1
		+1
\	Without	+1
		+1
\	Without	+1
		+1
\	Without	+1
		+1
\	Without	+1
		+1
\	Without	+1
		+1
Y. W.	With	+2
		+1
\	Without	+1
		+1
\	Without	+1
		+1

Final Quality of Evidence (High, Moderate, Low, Very Low)														
	Low	Low	Low	Low	Low	Low	Low	Low	Low	Low	Very Low	Low	Low	Low

(continued Table S6)

Study	Appraiser	Study Design		Initial Quality		Lower if					Higher if			With/Without Disagreement	Disagreement Adjudication by P. F.	Final Quality of Evidence
		RT	OS	High	Low	I	II	III	IV	V	I	II	III			
Crisan et al. 2016	S. F.		√		√	0	0	0	0	-1	0	0	+1	Without	\	Low
	Y. W.		√		√	0	0	0	0	-1	0	0	+1			
Cinar et al. 2019	S. F.		√		√	0	0	0	0	-1	0	0	+1	Without	\	Low
	Y. W.		√		√	0	0	0	0	-1	0	0	+1			
Tohi et al. 2019	S. F.		√		√	0	0	0	0	-1	0	0	+1	Without	\	Low
	Y. W.		√		√	0	0	0	0	-1	0	0	+1			
Tian et al. 2020	S. F.		√		√	0	0	0	0	-1	0	0	+1	Without	\	Low
	Y. W.		√		√	0	0	0	0	-1	0	0	+1			
Zhao et al. 2020	S. F.		√		√	-1	0	0	0	-1	0	0	+1	Without	\	Very Low
	Y. W.		√		√	-1	0	0	0	-1	0	0	+1			
Keranmu et al. 2021	S. F.		√		√	0	0	-1	0	-1	0	0	+1	Without	\	Very Low
	Y. W.		√		√	0	0	-1	0	-1	0	0	+1			
Liu et al. 2021	S. F.		√		√	0	0	0	0	-1	0	0	+1	Without	\	Low
	Y. W.		√		√	0	0	0	0	-1	0	0	+1			
	S. F.		√		√	-1	0	0	0	-1	0	0	+1	Without	\	Very Low

Liu et al. 2021	Y. W.	√	√	-1	0	0	0	-1	0	0	+1			
Ma et al. 2021	S. F.	√	√	0	0	0	0	-1	0	0	+1	Without	\	Low
	Y. W.	√	√	0	0	0	0	-1	0	0	+1			
Chen et al. 2023	S. F.	√	√	0	-1	0	0	-1	0	0	+1	Without	\	Low
	Y. W.	√	√	0	-1	0	0	-1	0	0	+1			
Scherñuk et al. 2023	S. F.	√	√	-1	0	0	-1	-1	0	0	+1	Without	\	Very Low
	Y. W.	√	√	-1	0	0	-1	-1	0	0	+1			
Zhang et al. 2023	S. F.	√	√	0	0	0	0	-1	0	0	+1	Without	\	Low
	Y. W.	√	√	0	0	0	0	-1	0	0	+1			

Notes: Appraisers of quality of evidence are S. F. - Shuyang Feng, Y. W. – Yiting Wu. Ping Fu (P. F.) made the decision to adopt the evaluation from one of appraisers as the final quality of evidence when disagreement arose.

Supplementary Table S7 – Quality of evidence evaluation among HALP-related studies included in the review evaluated by the Grading of Recommendations, Assessment, Development, and Evaluation (GRADE) system.

Study	Appraiser	Study Design		Initial Quality		Lower if					Higher if			With/Without Disagreement	Disagreement Adjudication by P. F.	Final Quality of Evidence
		RT	OS	High	Low	I	II	III	IV	V	I	II	III			
Desai et al. 2003	S. F.		√		√	0	0	0	0	-1	0	0	+1	Without	\	Low
	Y. W.		√		√	0	0	0	0	-1	0	0	+1			
Varkarakis et al. 2004	S. F.		√		√	0	0	0	0	-1	0	0	+1	Without	\	Low
	Y. W.		√		√	0	0	0	0	-1	0	0	+1			
Kapoor et al. 2006	S. F.		√		√	0	0	0	0	-1	0	0	+1	Without	\	Low
	Y. W.		√		√	0	0	0	0	-1	0	0	+1			
Henderson et al. 2008	S. F.		√		√	0	0	0	0	-1	0	0	+1	Without	\	Low
	Y. W.		√		√	0	0	0	0	-1	0	0	+1			
Martin et al. 2008	S. F.		√		√	0	0	0	0	-1	0	0	+1	Without	\	Low
	Y. W.		√		√	0	0	0	0	-1	0	0	+1			
Hoang et al. 2010	S. F.		√		√	0	0	0	0	-1	0	0	+1	Without	\	Low
	Y. W.		√		√	0	0	0	0	-1	0	0	+1			
Castillo et al. 2014	S. F.		√		√	0	0	0	0	-1	0	0	+2	With	Y. W.	Low
	Y. W.		√		√	0	0	0	0	-1	0	0	+1			
Tohi et al. 2019	S. F.		√		√	0	0	0	0	-1	0	0	+1	Without	\	Low
	Y. W.		√		√	0	0	0	0	-1	0	0	+1			

Notes: Appraisers of quality of evidence are S. F. - Shuyang Feng, Y. W. – Yiting Wu. Ping Fu (P.F.) made the decision to adopt the evaluation from one of appraisers as the final quality of evidence when disagreement arose.

SUPPLEMENTARY TABLE 2

Supplementary Table S8 – Quality of evidence evaluation among ROB-related studies included in the review evaluated by the Grading of Recommendations, Assessment, Development, and Evaluation (GRADE) system.

Study	Appraiser	Study Design		Initial Quality		Lower if					Higher if			With/Without Disagreement	Disagreement Adjudication by P. F.	Final Quality of Evidence
		RT	OS	High	Low	I	II	III	IV	V	I	II	III			
Abaza 2010	S. F.		√		√	0	0	0	0	-1	0	0	+1	Without	\	Low
	Y. W.		√		√	0	0	0	0	-1	0	0	+1			
Gill et al. 2015	S. F.		√		√	0	0	0	0	-1	0	0	+1	Without	\	Low
	Y. W.		√		√	0	0	0	0	-1	0	0	+1			
Wang et al. 2015	S. F.		√		√	0	0	0	0	-1	0	0	+1	Without	\	Low
	Y. W.		√		√	0	0	0	0	-1	0	0	+1			
Abaza et al. 2016	S. F.		√		√	0	0	0	0	-1	0	0	+1	Without	\	Low
	Y. W.		√		√	0	0	0	0	-1	0	0	+1			
Kundavaram et al. 2016	S. F.		√		√	0	0	0	0	-1	0	0	+1	Without	\	Low
	Y. W.		√		√	0	0	0	0	-1	0	0	+1			
Chopra et al. 2016	S. F.		√		√	0	0	0	0	-1	0	0	+1	Without	\	Low
	Y. W.		√		√	0	0	0	0	-1	0	0	+1			
Davila et al. 2016	S. F.		√		√	0	0	0	0	-1	0	0	+1	Without	\	Low
	Y. W.		√		√	-1	0	0	0	-1	0	0	+1			
Gu et al. 2017	S. F.		√		√	-1	0	0	0	-1	0	0	+1	Without	\	Very Low
	Y. W.		√		√	-1	0	0	0	-1	0	0	+1			
Wang et al. 2017	S. F.		√		√	0	0	0	0	-1	0	0	+2	With	Y.W.	Low
	Y. W.		√		√	0	0	0	0	-1	0	0	+1			
Ke et al. 2018	S. F.		√		√	0	0	0	0	-1	0	0	+1	Without	\	Low
	Y. W.		√		√	0	0	0	0	-1	0	0	+1			
Fan et al. 2019	S. F.		√		√	0	0	0	0	-1	0	0	+1	Without	\	Low
	Y. W.		√		√	0	0	0	0	-1	0	0	+1			
Rose et al. 2019	S. F.		√		√	-1	0	0	0	-1	0	0	+1	Without	\	Very Low
	Y. W.		√		√	-1	0	0	0	-1	0	0	+1			
Du et al. 2020	S. F.		√		√	0	0	0	0	-1	0	0	+1	Without	\	Low

	Y. W.	√	√	0	0	0	0	-1	0	0	+1			
Kishore et al.	S. F.	√	√	0	0	0	0	-1	0	0	+1	Without	\	Low
2020	Y. W.	√	√	0	0	0	0	-1	0	0	+1			
Shen et al.	S. F.	√	√	0	0	0	0	-1	0	0	+1	Without	\	Low
2020	Y. W.	√	√	0	0	0	0	-1	0	0	+1			
Shen et al.	S. F.	√	√	0	0	0	0	-1	0	0	+1	Without	\	Low
2020	Y. W.	√	√	0	0	0	0	-1	0	0	+1			
Shi et al.	S. F.	√	√	0	0	0	0	-1	0	0	+1	Without	\	Low
2020	Y. W.	√	√	0	0	0	0	-1	0	0	+1			
Ma et al.	S. F.	√	√	0	0	0	0	-1	0	0	+1	Without	\	Low
2021	Y. W.	√	√	0	0	0	0	-1	0	0	+1			
Wu et al.	S. F.	√	√	0	0	0	0	-1	0	0	+1	With	S.F.	Low
2021	Y. W.	√	√	0	-1	0	0	-1	0	0	+1			
Miyake et al.	S. F.	√	√	-2	0	0	0	0	0	0	+1	Without	\	Very Low
2022	Y. W.	√	√	-2	0	0	0	0	0	0	+1			
Morgan et al.	S. F.	√	√	0	0	0	0	-1	0	0	+1	Without	\	Low
2022	Y. W.	√	√	0	0	0	0	-1	0	0	+1			
Zhao et al.	S. F.	√	√	-1	0	0	0	-1	0	0	+1	Without	\	Very Low
2022	Y. W.	√	√	-1	0	0	0	-1	0	0	+1			
Zhang et al.	S. F.	√	√	0	0	0	0	-1	0	0	+1	Without	\	Low
2023	Y. W.	√	√	0	0	0	0	-1	0	0	+1			
Zhang et al.	S. F.	√	√	0	0	0	0	0	0	0	+1	Without	\	Moderate
2023	Y. W.	√	√	0	0	0	0	0	0	0	+1			

Notes: Appraisers of quality of evidence are S. F. - Shuyang Feng, Y. W. – Yiting Wu. Ping Fu (P.F.) made the decision to adopt the evaluation from one of appraisers as the final quality of evidence when disagreement arose.
