# Supplementary material for: Minimally Invasive Surgical Techniques for Renal Cell Carcinoma with Intravenous Tumor Thrombus: A Systematic Review of Laparoscopic and Robotic-Assisted Approaches
Source: Curr Oncol. 2025 Apr 28;32(5):256. doi: 10.3390/curroncol32050256 (PMC12109617; doi:10.3390/curroncol32050256)
Supplement: Supplementary file 1 [file curroncol-32-00256-s001.zip › curroncol-3545779-supplementary/Table for RoB (Tables S1-S5).pdf]

Supplementary Table S1 – Risk of bias assessment among LAP-related cohort studies included in the review evaluated by the Newcastle-Ottawa-Scale (NOS) for cohort studies.

Study (Author/Year)	Appraiser	Selection				Comparability	Outcome			With/Without Disagreement	Disagreement Adjudication by P. F.	Overall Assessment
		1	2	3	4	1	1	2	3			
Xu *et al.* 2014	S. F.<br>Y. W.	b *<br>b *	a *<br>a *	a *<br>a *	a *<br>a *	b *<br>b *	d<br>d	b<br>b	c<br>c	Without	\	M-5/9
Zhao et al. 2020	S. F.<br>Y. W.	c<br>c	a *<br>a *	a *<br>a *	a *<br>a *	a *<br>a *	d<br>d	b<br>b	c<br>c	Without	\	M-4/9
Liu et al. 2021	S. F.<br>Y. W.	c<br>c	a *<br>a *	a *<br>a *	a *<br>a *	a *<br>a *	b *<br>b *	a *<br>a *	a *<br>b *	Without	\	H-7/9
Liu et al. 2021	S. F.<br>Y. W.	c<br>c	a *<br>a *	a *<br>a *	a *<br>a *	a *<br>a *	b *<br>b *	b<br>a *	a *<br>a *	With	S. F.	M-6/9
Chen et al. 2023	S. F.<br>Y. W.	c<br>c	a *<br>a *	a *<br>a *	a *<br>a *	a *<br>a *	b *<br>b *	a *<br>a *	a *<br>a *	Without	\	H-7/9
Scherñuk et al. 2023	S. F.<br>Y. W.	c<br>c	a *<br>a *	a *<br>a *	a *<br>a *	a *<br>a *	b *<br>b *	b<br>b	d<br>d	Without	\	M-6/9
Zhang et al. 2023	S. F.<br>Y. W.	b *<br>b *	a *<br>a *	a *<br>a *	a *<br>a *	a *<br>a *	b *<br>b *	a *<br>a *	a *<br>a *	Without	\	H-9/9

Notes: *Overall Assessment* is evaluated by NOS recommended quality levels: L-Low quality (0-3 stars), M-Medium quality (4-6 stars), H-High quality (7-9 stars). Appraisers of risk of bias are S. F. - Shuyang Feng, Y. W. – Yiting Wu. Ping Fu (P.F.) made the decision to adopt the evaluation from one of appraisers as the overall assessment when disagreement arose.

Supplementary Table S2 – Risk of bias assessment among ROB-related cohort studies included in the review evaluated by the Newcastle-Ottawa-Scale (NOS) for cohort studies.

Study (Author/Year)	Appraiser	Selection				Comparability	Outcome			With/Without Disagreement	Disagreement Adjudication by P. F.	Overall Assessment
		1	2	3	4	1	1	2	3			
Gu *et al.* 2017	S. F.	b *	a *	a *	a *	b *	d	b	c	Without	\	M-5/9
	Y. W.	b *	a *	a *	a *	b *	d	b	c			
Rose et al. 2019	S. F.	c	a *	a *	a *	b *	b *	b	c	Without	\	M-5/9
	Y. W.	c	a *	a *	a *	b *	b *	b	c			
Wu et al. 2021	S. F.	c	a *	a *	a *	b *	b *	a *	a *	Without	\	H-8/9
	Y. W.	c	a *	a *	a *	b *	b *	a *	b *			
Zhao et al. 2023	S. F.	c	a *	a *	a *	b *	b *	a *	b *	Without	\	H-8/9
	Y. W.	c	a *	a *	a *	b *	b *	a *	b *			
Zhang et al. 2023	S. F.	b *	a *	a *	a *	b *	b *	a *	a *	Without	\	H-9/9
	Y. W.	b *	a *	a *	a *	b *	b *	a *	a *			

Notes: *Overall Assessment* is evaluated by NOS recommended quality levels: L-Low quality (0-3 stars), M-Medium quality (4-6 stars), H-High quality (7-9 stars). Appraisers of risk of bias are S. F. - Shuyang Feng, Y. W. – Yiting Wu. Ping Fu (P.F.) made the decision to adopt the evaluation from one of appraisers as the overall assessment when disagreement arose.

Supplementary Table S3 – Risk of bias assessment among LAP-related case-series reports included in the review evaluated by the IHE-Delphi-Tool for case-series studies.

Study	Appraiser	Study Objective (SO)	Study Population (SP)				
		1	2	3	4	5	6
Desai *et al.* 2003	S. F.	Y	Y	Y	Y	N	Y
	Y. W.	Y	Y	Y	Y	N	Y
Kapoor *et al.*	S. F.	Y	Y	Y	Y	N	Y
	Y. W.	Y	Y	Y	Y	N	Y
Hammond *et al.*	S. F.	Y	Y	N	Y	N	Y
	Y. W.	Y	Y	N	Y	N	Y
Steinnerd *et al.*	S. F.	Y	Y	N	Y	N	Y
	Y. W.	Y	Y	N	Y	N	Y
Martin *et al.* 2008	S. F.	Y	Y	N	Y	N	Y
	Y. W.	Y	Y	N	Y	N	Y
Guzzo *et al.* 2009	S. F.	Y	Y	N	Y	N	Y
	Y. W.	Y	Y	N	Y	N	Y
Liss *et al.* 2013	S. F.	Y	Y	Y	Y	Y	Y
	Y. W.	Y	Y	Y	Y	Y	Y
Bansal *et al.* 2014	S. F.	Y	Y	Y	Y	N	Y
	Y. W.	Y	Y	Y	Y	N	Y
Wang *et al.* (Left)	S. F.	Y	Y	N	Y	N	Y
	Y. W.	Y	Y	N	Y	N	Y
Wang *et al.*	S. F.	Y	Y	N	Y	N	Y
	Y. W.	Y	Y	N	Y	N	Y
Castillo *et al.* 2014	S. F.	Y	Y	N	Y	N	Y
	Y. W.	Y	Y	N	Y	N	Y
Shao *et al.* 2015	S. F.	Y	Y	N	Y	N	Y
	Y. W.	Y	Y	N	Y	N	Y
Wang *et al.* 2016	S. F.	Y	Y	N	Y	N	Y
	Y. W.	Y	Y	N	Y	N	Y
Crisan *et al.* 2018	S. F.	Y	Y	N	Y	Y	Y
	Y. W.	Y	Y	N	Y	Y	Y
Cinar *et al.* 2019	S. F.	Y	Y	N	Y	N	Y
	Y. W.	Y	Y	N	Y	N	Y
Tohi *et al.* 2019	S. F.	Y	Y	N	Y	N	Y
	Y. W.	Y	Y	N	Y	N	Y
Tian *et al.* 2020	S. F.	Y	Y	N	Y	N	Y
	Y. W.	Y	Y	N	Y	N	Y
Keramnu *et al.*	S. F.	Y	Y	N	Y	N	Y
	Y. W.	Y	Y	N	Y	N	Y
Ma *et al.* 2021	S. F.	Y	Y	N	Y	N	Y
	Y. W.	Y	Y	N	Y	N	Y

Statistical Analysis (SA)	Outcome Measures (O)			Intervention and Co-intervention (I)	
12	11	10	9	8	7
Y	N	Y	Y	\	Y
Y	N	Y	Y	\	Y
Y	N	Y	Y	Y	Y
Y	N	Y	Y	Y	Y
Y	N	Y	Y	\	Y
Y	N	Y	Y	\	Y
Y	N	Y	Y	\	Y
Y	N	Y	Y	\	Y
Y	N	Y	Y	Y	Y
Y	N	Y	Y	Y	Y
Y	N	Y	Y	\	Y
Y	N	Y	Y	\	Y
Y	N	Y	Y	Y	Y
Y	N	Y	Y	Y	Y
Y	N	Y	Y	\	Y
Y	N	Y	Y	\	Y
Y	N	Y	Y	\	Y
Y	N	Y	Y	\	Y
Y	N	Y	Y	\	Y
Y	N	Y	Y	\	Y
Y	N	Y	Y	Y	Y
Y	N	Y	Y	Y	Y
Y	N	Y	Y	Y	Y
Y	N	Y	Y	Y	Y
Y	N	Y	Y	\	Y
Y	N	Y	Y	\	Y
Y	Y	Y	Y	\	Y
Y	Y	Y	Y	Y	Y
Y	N	Y	Y	\	Y
Y	N	Y	Y	\	Y
Y	N	Y	Y	Y	Y
Y	N	Y	Y	Y	Y
Y	N	Y	Y	\	Y
Y	N	Y	Y	\	Y
Y	N	Y	Y	\	Y
Y	N	Y	Y	\	Y
Y	Y	Y	Y	Y	Y
Y	Y	Y	Y	Y	Y

Competing Interests and Source of Support (CI)	Results and Conclusions (R)				
18	17	16	15	14	13
N	Y	Y	Y	N	Y
N	Y	Y	Y	N	Y
N	Y	Y	Y	Y	Y
N	Y	Y	Y	Y	Y
N	Y	Y	N	Y	Y
N	Y	Y	N	Y	Y
N	Y	Y	Y	Y	Y
N	Y	Y	Y	Y	Y
N	Y	Y	Y	Y	Y
N	Y	Y	Y	Y	Y
Y	Y	Y	Y	Y	Y
Y	Y	Y	Y	Y	Y
Y	Y	Y	Y	Y	Y
Y	Y	Y	Y	Y	Y
Y	Y	Y	Y	Y	Y
Y	Y	Y	Y	Y	Y
Y	Y	Y	N	Y	Y
Y	Y	Y	N	Y	Y
Y	Y	Y	N	Y	Y
Y	Y	Y	N	Y	Y
N	Y	Y	Y	Y	Y
N	Y	Y	Y	Y	Y
Y	Y	Y	N	Y	Y
Y	Y	Y	N	Y	Y
Y	Y	Y	N	Y	Y
Y	Y	Y	N	Y	Y
Y	Y	Y	Y	Y	Y
Y	Y	Y	Y	Y	Y
Y	Y	Y	Y	Y	Y
Y	Y	Y	Y	Y	Y
Y	Y	Y	N	Y	Y
Y	Y	Y	N	Y	Y
Y	Y	Y	Y	Y	Y
Y	Y	Y	Y	Y	Y
Y	Y	Y	Y	Y	Y
Y	Y	Y	Y	Y	Y
Y	Y	N	Y	Y	Y
Y	Y	N	Y	Y	Y

Overall Assessment	Disagreement Adjudication by P. F.	With/Without Disagreement
Acceptable (13/17)	/	Without
Acceptable (15/18)	/	Without
Acceptable (13/17)	/	Without
Acceptable (13/17)	/	Without
Acceptable (14/18)	/	Without
Acceptable (14/17)	/	Without
Acceptable (17/18)	/	Without
Acceptable (15/17)	/	Without
Acceptable (13/17)	/	Without
Acceptable (13/17)	/	Without
Acceptable (14/18)	/	Without
Acceptable (14/18)	/	Without
Acceptable (13/17)	/	Without
Acceptable (17/18)	Y. W.	With
Acceptable (14/17)	/	Without
Acceptable (14/18)	/	Without
Acceptable (14/17)	/	Without
Acceptable (14/17)	/	Without
Acceptable (15/18)	/	Without

Notes: *Overall Assessment* is evaluated by the recommendation from the IHE board of directors: "A study with 14 or more yes responses (≥70%) was considered to be of acceptable quality". Appraisers of risk of bias are S. F. - Shuyang Feng, Y. W. – Yiting Wu. Ping Fu (P.F.) made the decision to adopt the evaluation from one of appraisers as the overall assessment when disagreement arose.

Supplementary Table S4 – Risk of bias assessment among HALP-related case-series reports included in the review evaluated by the IHE-Delphi-Tool for case-series studies.

Study	Appraiser	SO	SP					I		O			SA	R					CI	With/Without Disaggreement	Disagreement Adjudication by P. F.	Overall Assessment
		1	2	3	4	5	6	7	8	9	10	11	12	13	14	15	16	17	18			
Varkarakis et al.	S. F.	Y	Y	N	Y	N	Y	Y	Y	Y	Y	N	Y	Y	Y	N	Y	Y	N	Without	\	Acceptable
2004	Y. W.	Y	Y	N	Y	N	Y	Y	Y	Y	Y	N	Y	Y	Y	N	Y	Y	N			(13/18)
Kapoor et al.	S. F.	Y	Y	Y	Y	N	Y	Y	Y	Y	Y	N	Y	Y	Y	Y	Y	Y	N	Without	\	Acceptable
2006	Y. W.	Y	Y	Y	Y	N	Y	Y	Y	Y	Y	N	Y	Y	Y	Y	Y	Y	N			(15/18)
Henderson et al.	S. F.	Y	Y	Y	Y	N	Y	Y	Y	Y	Y	N	Y	Y	Y	Y	Y	Y	N	Without	\	Acceptable
2008	Y. W.	Y	Y	Y	Y	N	Y	Y	Y	Y	Y	N	Y	Y	Y	Y	Y	Y	N			(15/18)
Martin et al.	S. F.	Y	Y	N	Y	N	Y	Y	Y	Y	Y	N	Y	Y	Y	Y	Y	Y	N	Without	\	Acceptable
2008	Y. W.	Y	Y	N	Y	N	Y	Y	Y	Y	Y	N	Y	Y	Y	Y	Y	Y	N			(14/18)
Hoang et al.	S. F.	Y	Y	N	Y	N	Y	Y	Y	Y	Y	N	Y	Y	Y	Y	Y	Y	Y	With	S.F.	Acceptable
2010	Y. W.	Y	Y	N	Y	N	Y	Y	Y	Y	Y	N	Y	Y	Y	N	Y	Y	Y			(15/18)
Castillo et al.	S. F.	Y	Y	N	Y	N	Y	Y	Y	Y	Y	N	Y	Y	Y	Y	Y	Y	N	Without	\	Acceptable
2014	Y. W.	Y	Y	N	Y	N	Y	Y	Y	Y	Y	N	Y	Y	Y	Y	Y	Y	N			(14/18)
Tohi et al.	S. F.	Y	Y	N	Y	N	Y	Y	Y	Y	Y	N	Y	Y	Y	N	Y	Y	Y	Without	\	Acceptable
2019	Y. W.	Y	Y	N	Y	N	Y	Y	Y	Y	Y	N	Y	Y	Y	N	Y	Y	Y			(14/18)

Notes: *Overall Assessment* is evaluated by the recommendation from the IHE board of directors: "A study with 14 or more yes responses (≥70%) was considered to be of acceptable quality". Appraisers of risk of bias are S. F. - Shuyang Feng, Y. W. – Yiting Wu. Ping Fu (P.F.) made the decision to adopt the evaluation from one of appraisers as the overall assessment when disagreement arose.

Supplementary Table S5 – Risk of bias assessment among ROB-related case-series reports included in the review evaluated by the IHE-Delphi-Tool for case-series studies.

Study	Appraiser	SO	SP					I		O			SA	R					CI	With/Without Disaggrement	Disagreement Adjudication by P. F.	Overall Assessment
		1	2	3	4	5	6	7	8	9	10	11	12	13	14	15	16	17	18			
Abaza 2010	S. F.	Y	Y	N	Y	N	Y	Y	\	Y	Y	N	Y	Y	Y	Y	Y	Y	Y	Without	\	Acceptable
	Y. W.	Y	Y	N	Y	N	Y	Y	\	Y	Y	N	Y	Y	Y	Y	Y	Y	Y			(14/17)
Gill et al.	S. F.	Y	Y	N	Y	N	Y	Y	\	Y	Y	Y	Y	Y	Y	Y	Y	Y	N	Without	\	Acceptable
2015	Y. W.	Y	Y	N	Y	N	Y	Y	\	Y	Y	Y	Y	Y	Y	Y	Y	Y	N			(14/17)
Wang et al.	S. F.	Y	Y	N	Y	N	Y	Y	Y	Y	Y	Y	Y	Y	Y	Y	Y	Y	Y	Without	\	Acceptable
2015	Y. W.	Y	Y	N	Y	N	Y	Y	Y	Y	Y	Y	Y	Y	Y	Y	Y	Y	Y			(16/18)
Abaza et al.	S. F.	Y	Y	Y	Y	Y	Y	Y	\	Y	Y	N	Y	Y	N	Y	Y	Y	N	Without	\	Acceptable
2016	Y. W.	Y	Y	Y	Y	Y	Y	Y	\	Y	Y	N	Y	Y	N	Y	Y	Y	N			(14/17)
Kundavaram	S. F.	Y	Y	N	Y	N	Y	Y	Y	Y	Y	N	Y	Y	Y	Y	Y	Y	Y	Without	\	Acceptable
et al. 2016	Y. W.	Y	Y	N	Y	N	Y	Y	Y	Y	Y	N	Y	Y	Y	Y	Y	Y	Y			(15/18)
Chopra et al.	S. F.	Y	Y	Y	Y	Y	Y	Y	\	Y	Y	N	Y	Y	Y	Y	Y	Y	Y	Without	\	Acceptable
2016	Y. W.	Y	Y	Y	Y	Y	Y	Y	\	Y	Y	N	Y	Y	Y	Y	Y	Y	Y			(16/17)
Davila et al.	S. F.	Y	Y	N	Y	N	Y	Y	Y	Y	Y	N	N	Y	N	Y	Y	Y	Y	Without	\	Acceptable
2016	Y. W.	Y	Y	N	Y	N	Y	Y	Y	Y	Y	N	N	Y	N	Y	Y	Y	Y			(13/18)
Wang et al.	S. F.	Y	Y	N	Y	N	Y	Y	Y	Y	Y	Y	Y	Y	Y	Y	Y	Y	Y	With	Y. W.	Acceptable
2017	Y. W.	Y	Y	N	Y	N	Y	Y	\	Y	Y	Y	Y	Y	Y	Y	Y	Y	Y			(15/17)
Ke et al. 2018	S. F.	Y	Y	N	Y	N	Y	Y	\	Y	Y	Y	Y	Y	N	Y	Y	Y	Y	Without	\	Acceptable
	Y. W.	Y	Y	N	Y	N	Y	Y	\	Y	Y	Y	Y	Y	N	Y	Y	Y	Y			(14/17)
Fan et al.	S. F.	Y	Y	N	Y	N	Y	Y	\	Y	Y	N	Y	Y	Y	Y	Y	Y	Y	Without	\	Acceptable
2019	Y. W.	Y	Y	N	Y	N	Y	Y	\	Y	Y	N	Y	Y	Y	Y	Y	Y	Y			(14/17)
Du et al. 2020	S. F.	Y	Y	N	Y	N	Y	Y	\	Y	Y	Y	Y	Y	N	Y	Y	Y	N	Without	\	Acceptable
	Y. W.	Y	Y	N	Y	N	Y	Y	\	Y	Y	Y	Y	Y	N	Y	Y	Y	N			(13/17)
Kishore et al.	S. F.	Y	Y	Y	Y	N	Y	Y	\	Y	Y	Y	Y	Y	Y	Y	Y	Y	N	Without	\	Acceptable
2020	Y. W.	Y	Y	Y	Y	N	Y	Y	\	Y	Y	Y	Y	Y	Y	Y	Y	Y	N			(15/17)
Shen et al.	S. F.	Y	Y	N	Y	Y	Y	Y	Y	Y	Y	Y	Y	Y	Y	Y	Y	Y	Y	Without	\	Acceptable
2020	Y. W.	Y	Y	N	Y	Y	Y	Y	Y	Y	Y	Y	Y	Y	Y	Y	Y	Y	Y			(17/18)
	S. F.	Y	Y	N	Y	N	Y	Y	\	Y	Y	N	Y	Y	N	Y	Y	Y	Y	With	S. F.	

Shen et al. 2020	Y. W.	Y	Y	N	Y	N	Y	Y	Y	Y	Y	N	Y	Y	N	Y	Y	Y	Y			Acceptable (13/17)
Shi et al. 2020	S. F.	Y	Y	Y	Y	N	Y	Y	\	Y	Y	N	Y	Y	Y	Y	Y	Y	Y	Without	\	Acceptable (15/17)
	Y. W.	Y	Y	Y	Y	N	Y	Y	\	Y	Y	N	Y	Y	Y	Y	Y	Y	Y			
Ma et al. 2021	S. F.	Y	Y	N	Y	N	Y	Y	\	Y	Y	N	Y	Y	Y	Y	Y	Y	N	Without	\	Acceptable (13/17)
	Y. W.	Y	Y	N	Y	N	Y	Y	\	Y	Y	N	Y	Y	Y	Y	Y	Y	N			
Miyake et al. 2022	S. F.	Y	Y	N	Y	N	Y	N	\	Y	Y	N	N	N	N	N	N	N	Y	Without	\	Unacceptable (7/17)
	Y. W.	Y	Y	N	Y	N	Y	N	\	Y	Y	N	N	N	N	N	N	N	Y			
Morgan et al. 2022	S. F.	Y	Y	N	Y	N	Y	Y	\	Y	Y	Y	Y	Y	Y	Y	Y	Y	N	Without	\	Acceptable (14/17)
	Y. W.	Y	Y	N	Y	N	Y	Y	\	Y	Y	Y	Y	Y	Y	Y	Y	Y	N			
Zhao et al. 2022	S. F.	Y	Y	N	Y	N	Y	Y	\	Y	Y	N	Y	Y	Y	Y	N	Y	Y	Without	\	Acceptable (13/17)
	Y. W.	Y	Y	N	Y	N	Y	Y	\	Y	Y	N	Y	Y	Y	Y	N	Y	Y			

Notes: *Overall Assessment* is evaluated by the recommendation from the IHE board of directors: “A study with 14 or more yes responses (≥70%) was considered to be of acceptable quality”. Appraisers of risk of bias are S. F. - Shuyang Feng, Y. W. – Yiting Wu. Ping Fu (P.F.) made the decision to adopt the evaluation from one of appraisers as the overall assessment when disagreement arose.
