# Supplementary material for: Minimally Invasive Surgical Techniques for Renal Cell Carcinoma with Intravenous Tumor Thrombus: A Systematic Review of Laparoscopic and Robotic-Assisted Approaches
Source: Curr Oncol. 2025 Apr 28;32(5):256. doi: 10.3390/curroncol32050256 (PMC12109617; doi:10.3390/curroncol32050256)
Supplement: Supplementary file 1 [file curroncol-32-00256-s001.zip › curroncol-3545779-supplementary/text file for Search Strategy.pdf]

Search Strategy

PROSPERO ID: 210171

Title: Outcomes and complications of laparoscopic vs robotic surgery for locally advanced renal cell carcinoma with intravenous tumor thrombus: a systematic review and meta-analysis

Named contact: Shuyang Feng

Keywords: renal cell carcinoma, kidney neoplasm, intravenous tumor thrombus, thrombosis, laparoscopy, hand-assisted laparoscopy, robotic surgical procedures

Notes: We choose the retrieval process on MEDLINE (via PubMed) to demonstrate the search strategy of the review. Additionally, we will be very grateful for your patience to browse the whole process!

#1 Search (((Carcinomas, Renal Cell[Title/Abstract]) OR (Renal Cell Carcinomas[Title/Abstract])) OR (Adenocarcinoma, Renal Cell[Title/Abstract])) OR (Adenocarcinomas, Renal Cell[Title/Abstract])) OR (Renal Cell Adenocarcinoma[Title/Abstract])) OR (Renal Cell Adenocarcinomas[Title/Abstract])) OR (Adenocarcinoma Of Kidney[Title/Abstract])) OR (Adenocarcinoma Of Kidneys[Title/Abstract])) OR (Kidney, Adenocarcinoma Of[Title/Abstract])) OR (Kidneys, Adenocarcinoma Of[Title/Abstract])) OR (Renal Cell Cancer[Title/Abstract])) OR (Cancer, Renal Cell[Title/Abstract])) OR (Cancers, Renal Cell[Title/Abstract])) OR (Renal Cell Cancers[Title/Abstract])) OR (Adenocarcinoma, Renal[Title/Abstract])) OR (Adenocarcinomas, Renal[Title/Abstract])) OR (Renal Adenocarcinoma[Title/Abstract])) OR (Renal Adenocarcinomas[Title/Abstract])) OR (Nephroid Carcinoma[Title/Abstract])) OR (Carcinoma, Nephroid[Title/Abstract])) OR (Carcinomas, Nephroid[Title/Abstract])) OR (Nephroid Carcinomas[Title/Abstract])) OR (Renal Cell Carcinoma[Title/Abstract])) OR (Chromophobe Renal Cell

Carcinoma[Title/Abstract])) OR (Sarcomatoid Renal Cell Carcinoma[Title/Abstract])) OR (Papillary Renal Cell Carcinoma[Title/Abstract])) OR (Renal Cell Carcinoma, Papillary[Title/Abstract])) OR (Chromophil Renal Cell Carcinoma[Title/Abstract])) OR (Clear Cell Renal Cell Carcinoma[Title/Abstract])) OR (Grawitz Tumor[Title/Abstract])) OR (Tumor, Grawitz[Title/Abstract])) OR (Clear Cell Renal Carcinoma[Title/Abstract])) OR (Carcinoma, Hypernephroid[Title/Abstract])) OR (Carcinomas, Hypernephroid[Title/Abstract])) OR (Hypernephroid Carcinoma[Title/Abstract])) OR (Hypernephroid Carcinomas[Title/Abstract])) OR (Hypernephroma[Title/Abstract])) OR (Hypernephromas[Title/Abstract])) OR (Collecting Duct Carcinoma (Kidney)[Title/Abstract])) OR (Carcinoma, Collecting Duct (Kidney)[Title/Abstract])) OR (Carcinomas, Collecting Duct (Kidney)[Title/Abstract])) OR (Collecting Duct Carcinomas (Kidney)[Title/Abstract])) OR (Collecting Duct Carcinoma of the Kidney[Title/Abstract])) OR (Renal Collecting Duct Carcinoma[Title/Abstract])) OR (Collecting Duct Carcinoma[Title/Abstract])) OR (Carcinoma, Collecting Duct[Title/Abstract])) OR (Carcinomas, Collecting Duct[Title/Abstract])) OR (Collecting Duct Carcinomas[Title/Abstract])

#2 Search "Carcinoma, Renal Cell"[Mesh]

#3 #1 OR #2

#4 Search (((((((((((((((Kidney Neoplasm[Title/Abstract]) OR (Neoplasm, Kidney[Title/Abstract])) OR (Renal Neoplasms[Title/Abstract])) OR (Neoplasm, Renal[Title/Abstract])) OR (Neoplasms, Renal[Title/Abstract])) OR (Renal Neoplasm[Title/Abstract])) OR (Neoplasms, Kidney[Title/Abstract])) OR (Cancer of Kidney[Title/Abstract])) OR (Kidney Cancers[Title/Abstract])) OR (Renal Cancer[Title/Abstract])) OR (Cancer, Renal[Title/Abstract])) OR (Cancers, Renal[Title/Abstract])) OR (Renal Cancers[Title/Abstract])) OR (Cancer of the Kidney[Title/Abstract])) OR (Kidney Cancer[Title/Abstract])) OR (Cancer, Kidney[Title/Abstract])) OR (Cancers, Kidney[Title/Abstract])

#5 Search "Kidney Neoplasms"[Mesh]

#6 #4 OR #5

#7 #3 OR #6

#8 Search (((((((intravenous tumor thrombus[Title/Abstract]) OR (venous tumor thrombus[Title/Abstract])) OR (vena cava thrombus[Title/Abstract]))

OR (vena cava thrombectomy[Title/Abstract])) OR (inferior vena cava thrombosis[Title/Abstract])) OR (inferior vena cava thrombus[Title/Abstract])) OR (tumor thrombus[Title/Abstract])) OR (IVC[Title/Abstract])

#9 Search (((((Thromboses[Title/Abstract]) OR (Thrombus[Title/Abstract])) OR (Blood Clot[Title/Abstract])) OR (Blood Clots[Title/Abstract])) OR (Clot, Blood[Title/Abstract])) OR (Clots, Blood[Title/Abstract])

#10 Search "Thrombosis"[Mesh]

#11 #8 OR #9 OR #10

#12 Search (((((((((((((((Laparoscopies[Title/Abstract]) OR (Celioscopy[Title/Abstract])) OR (Celioscopies[Title/Abstract])) OR (Peritoneoscopy[Title/Abstract])) OR (Peritoneoscopies[Title/Abstract])) OR (Surgical Procedures, Laparoscopic[Title/Abstract])) OR (Laparoscopic Surgical Procedure[Title/Abstract])) OR (Procedure, Laparoscopic Surgical[Title/Abstract])) OR (Procedures, Laparoscopic Surgical[Title/Abstract])) OR (Surgery, Laparoscopic[Title/Abstract])) OR (Laparoscopic Surgical Procedures[Title/Abstract])) OR (Laparoscopic Surgery[Title/Abstract])) OR (Laparoscopic Surgeries[Title/Abstract])) OR (Surgeries, Laparoscopic[Title/Abstract])) OR (Laparoscopic Assisted Surgery[Title/Abstract])) OR (Laparoscopic Assisted Surgeries[Title/Abstract])) OR (Surgeries, Laparoscopic Assisted[Title/Abstract])) OR (Surgery, Laparoscopic Assisted[Title/Abstract])) OR (Surgical Procedure, Laparoscopic[Title/Abstract])

#13 Search "Laparoscopy"[Mesh]

#14 Search (((((((((((Hand Assisted Laparoscopy[Title/Abstract]) OR (Hand-Assisted Laparoscopies[Title/Abstract])) OR (Laparoscopies, Hand-Assisted[Title/Abstract])) OR (Laparoscopy, Hand-Assisted[Title/Abstract])) OR (Hand-Assisted Laparoscopic Surgery[Title/Abstract])) OR (Hand Assisted Laparoscopic Surgery[Title/Abstract])) OR (Hand-Assisted Laparoscopic Surgeries[Title/Abstract])) OR (Laparoscopic Surgeries, Hand-Assisted[Title/Abstract])) OR (Laparoscopic Surgery, Hand-Assisted[Title/Abstract])) OR (Surgeries, Hand-Assisted Laparoscopic[Title/Abstract])) OR (Surgery, Hand-Assisted Laparoscopic[Title/Abstract])) OR (Hand-Assisted Laparoscopic Surgical Procedures[Title/Abstract])) OR (Hand Assisted Laparoscopic Surgical Procedures[Title/Abstract])

#15 Search "Hand-Assisted Laparoscopy"[Mesh]

#16 #12 OR #13 OR #14 OR #15

#17 Search (((((((((((((Procedure, Robotic Surgical[Title/Abstract]) OR (Procedures, Robotic Surgical[Title/Abstract])) OR (Robotic Surgical Procedure[Title/Abstract])) OR (Surgical Procedure, Robotic[Title/Abstract])) OR (Surgical Procedures, Robotic[Title/Abstract])) OR (Robot-Enhanced Procedures[Title/Abstract])) OR (Procedure, Robot-Enhanced[Title/Abstract])) OR (Procedures, Robot-Enhanced[Title/Abstract])) OR (Robot Enhanced Procedures[Title/Abstract])) OR (Robot-Enhanced Procedure[Title/Abstract])) OR (Robot-Enhanced Surgery[Title/Abstract])) OR (Robot Enhanced Surgery[Title/Abstract])) OR (Robot-Enhanced Surgeries[Title/Abstract])) OR (Surgeries, Robot-Enhanced[Title/Abstract])) OR (Surgery, Robot-Enhanced[Title/Abstract])

#18 Search "Robotic Surgical Procedures"[Mesh]

#19 #17 OR #18

#20 #7 AND #11 AND #16

#21 #7 AND #11 AND #19

#22 #20 OR #21
